# Ubiquitin‐specific protease 25 improves myocardial ischemia–reperfusion injury by deubiquitinating NLRP3 and negatively regulating NLRP3 inflammasome activity in cardiomyocytes

**DOI:** 10.1002/ctm2.70243

**Published:** 2025-02-22

**Authors:** Bozhi Ye, Diyun Xu, Lingfeng Zhong, Yi Wang, Wei Wang, Haowen Xu, Xue Han, Julian Min, Gaojun Wu, Wenhai Huang, Guang Liang

**Affiliations:** ^1^ Department of Pharmacy and Institute of Inflammation, Zhejiang Provincial People's Hospital, Affiliated People's Hospital Hangzhou Medical College Hangzhou Zhejiang China; ^2^ School of Pharmaceutical Sciences Hangzhou Medical College Hangzhou Zhejiang China; ^3^ Department of Cardiology and the Key Laboratory of Cardiovascular Disease of Wenzhou, the First Affiliated Hospital Wenzhou Medical University Wenzhou Zhejiang China; ^4^ Chemical Biology Research Center, School of Pharmaceutical Sciences Wenzhou Medical University Wenzhou Zhejiang China; ^5^ School of Pharmaceutical Sciences Hangzhou Normal University Hangzhou Zhejiang China; ^6^ Affiliated Yongkang First People's Hospital Hangzhou Medical College Yongkang Zhejiang China

**Keywords:** cardiomyocyte, myocardial ischemia–reperfusion injury, NLRP3, pyroptosis, ubiquitin‐specific protease 25

## Abstract

**Background:**

Myocardial ischemia/reperfusion injury (MI/RI) restricts the effect of myocardial reperfusion therapy and lacks effective prevention and treatment methods. Deubiquitinating enzymes (DUBs), especially members of the ubiquitin‐specific protease (USP) family of DUBs, are key proteins in the ubiquitination modification process and play a vital role in MI/RI. Therefore, we aimed to investigate the role of USP25, as a member of the USP family, in MI/RI and its molecular mechanism.

**Methods:**

Transcriptome sequencing was applied to evaluate the differential expression of USP families during hypoxia/reoxygenation (H/R) and validated in human and mouse heart samples and cardiomyocytes by performing quantitative polymerase chain reaction. Wild‐type or USP25^−/−^ mice were used to develop the MI/RI model. Co‐immunoprecipitation (Co‐IP) combined with liquid chromatography–tandem mass spectrometry analysis was used to screen the potential substrate protein of USP25 in H/R‐induced cardiomyocyte injury. TUNEL and Hoechst/propidium iodide staining and western blot were used to detect the level of pyroptosis. In addition, cardiomyocyte‐specific USP25 overexpression in NLRP3^−/−^ mice with AAV9 vectors was used to validate the biological function of USP25 and NLRP3 interaction.

**Results:**

We found that the expression level of USP25 was significantly decreased in I/R‐induced mouse heart tissues and primary cardiomyocytes in a time‐dependent manner. USP25 deficiency exacerbated MI/RI and aggravated I/R‐induced cardiac remodelling in mice. Mechanistically, USP25 directly binds to NLRP3 protein and K63‐linkedly deubiquitinates NLRP3 at residue K243 via its active site C178, thus hindering NLRP3–ASC interaction and ASC oligomerization to inhibit NLRP3 activation and pyroptosis in cardiomyocytes. We further showed that the overexpression of USP25 in cardiomyocytes ameliorated MI/RI in mice, whereas this protective effect disappeared when NLRP3 is knocked out.

**Conclusions:**

Our study demonstrated that USP25 ameliorates MI/RI by regulating NLRP3 activation and its mediated pyroptosis. This finding extends the protective role of USP25 in cardiovascular disease and provides an experimental basis for future USP25‐based drug development for the treatment of MI/RI.

**Key points:**

- The deubiquitinating enzyme USP25 was down‐regulated both in myocardial ischemia/reperfusion injury (MI/RI) myocardium tissues.
- The deficiency of USP25 worsened exacerbated MI/RI in mice, whereas the overexpression of USP25 in cardiomyocytes mitigated this pathological phenotype.
- USP25 directly interacts with the NLRP3 protein and deubiquitinates it via K63 linkage at residue K243 through its active site C178, thus affecting NLRP3‐ASC interaction and ASC oligomerization to inhibit NLRP3 activation and pyroptosis in cardiomyocytes.

## Graphical abstract

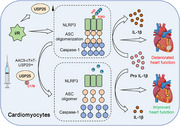

**ABBREVIATIONS**
AAR: area at risk
Co‐IP: co‐immunoprecipitation
DUBs: deubiquitinating enzymes
GSDMD: gasdermin D
H/R: hypoxia/reoxygenation
I/R: ischemia–reperfusion
INF: infarcted area size
MI/RI: myocardial ischaemia–reperfusion injury
NRPCs: neonatal rat primary cardiomyocytes
USP25: ubiquitin‐specific protease 25
WT: wild‐type

## 1 INTRODUCTION

Acute myocardial infarction, as a disease with the highest mortality and disability rate in the world, has become a serious problem threatening the life and health of people globally.^1^ Although timely and effective reperfusion therapy can save dying cardiomyocytes, restoring blood supply to ischaemic heart tissue can cause further damage, a phenomenon commonly referred to as myocardial ischemia–reperfusion injury (MI/RI).^2^ Current studies have shown that MI/RI is closely related to oxidative stress, endoplasmic reticulum stress, energy metabolism disorder and cardiomyocyte apoptosis/pyroptosis. In clinical practice, there is still a lack of effective intervention methods to prevent and treat myocardial reperfusion injury.^3^ Therefore, it is urgent to elucidate the molecular mechanism in the pathological progress of MI/RI and identify new and effective therapeutic targets for the treatment of this disease.

As non‐regenerative cells, it is very important for cardiomyocytes under various stimuli to maintain or remodel the integrity of cellular function through protein regulation mechanisms. Our group intends to explore the regulatory mechanism during myocardial I/R from the perspective of protein post‐translational modification. Ubiquitination/Deubiquitination is a vital type of protein post‐translational modification, which regulates the stability, intracellular localization or activity of the target protein.^4^ The process of deubiquitination is regulated by deubiquitinating enzymes (DUBs), which remove ubiquitin molecules from the substrate protein.^5^ So far, more than 100 DUBs have been identified in humans, comprising 7 families, among which the ubiquitin‐specific protease (USP) family is the largest one.^6^ Understanding the regulatory mechanism of DUBs in cardiac pathophysiology is expected to provide therapeutic strategies for related diseases.

Our team engages in the DUB study in cardiovascular diseases. Studies have indicated that this family is implicated in I/R injury. For instance, USP47 has been shown to promote apoptosis in rat cardiomyocytes following I/R injury by activating NF‐κB.^7^ Additionally, USP7 enhances ferroptosis by activating the p53/TfR1 pathway after I/R in the rat heart.^8^ Consequently, we postulate that the USP family plays a critical role in MI/RI and represents a potential molecular library for developing therapeutic agents aimed at treating MI/RI. Next, we have examined the gene expression profile of USP family members in heart tissues of MI/RI mice and identified the significantly decreased level of a DUB, USP25, indicating the potential involvement of USP25 in the pathogenesis of MI/RI. USP25 has been recently reported to play a role in various diseases,^9^ including diabetic nephropathy,^10^ ischaemic stroke,^11^ sepsis,^12^ viral infection,^13^ pancreatitis^14^ and Alzheimer's disease.^15^ In addition, USP25 is also a key regulator of cancer and plays vital roles in the pathological progression of colorectal cancer,^16^ liver cancer^17^ and pancreatic cancer.^18^ Recently, our group found that USP25 in cardiomyocytes negatively regulates pathological myocardial hypertrophy in hypertensive mice.^19^ However, the role of USP25 in MI/RI is unclear.

Therefore, further exploring the role of USP25 in MI/RI can systematically reveal its protective effect and enhance the potential of USP25 as a target for the treatment of heart diseases. In the present study, we showed that USP25 deletion exacerbated MI/RI and I/R‐induced cardiac remodelling, whereas overexpression of USP25 in cardiomyocytes ameliorated MI/RI in mice. We identified the nucleotide‐oligomerization domain‐like receptor (NLR) family pyrin domain–containing 3 (NLRP3) as the direct substrate of USP25. USP25 negatively regulates NLRP3 activity and inhibits pyroptosis in cardiomyocytes via K63‐linkedly deubiquitinating NLRP3 at K243 site. Our study illustrates a cardiomyocyte‐specific USP25–NLRP3 axis in regulating MI/RI and enhances the potential of USP25 as a target for the treatment of heart diseases.

## 2 METHODS

### 2.1 Animal experiments

The whole‐body USP25 knockout (USP25^−/−^) mice with a C57BL/6J background and wild‐type (WT) littermates were provided by Prof. Yuan from Tongji University. The whole‐body NLPR3 knockout (NLRP3^−/−^) mice with a C57BL/6J background (Strain No. VSM40005) and WT littermates were obtained from Beijing ViewSolid Biotech Co., Ltd. Mice were housed in a specific pathogen‐free environment with abundant food and water supply. Randomization was used when dividing the groups. All animal experiments were performed and analysed by a blinded experimenter.

Eight‐week‐old male USP25 or NLRP3 knockout mice and WT littermates with an average body weight of 22 ± 2 g were used to construct an MI/RI model. The left anterior descending (LAD) branch of the coronary artery was identified and ligated using a 7–0 silk suture approximately 2 mm distal to the LAD with a secure live knot. Successful ligation was confirmed by the observation of a pale colour at the distal edge of the ligature. Following 30 min of ischaemia, the suture was loosened to allow for reperfusion. The acute MI/RI model was induced by reperfusion for 4 h, whereas the chronic MI/RI model was established by reperfusion for 2 weeks. The sham‐operated group (Sham) underwent identical procedures as described above, with the exception of coronary artery ligation. No mice died during the MI/RI animal experiments. Upon completion of the modelling procedure, mice were euthanized under sodium pentobarbital anaesthesia. Heart tissue samples from infarcted regions of the mouse myocardium as well as serum samples were collected for subsequent experimental analysis.

To overexpress USP25 specifically in cardiomyocytes, we infected male mice with adeno‐associated virus serotype 9 that included a cardiac‐specific promoter cTNT (AAV9‐cTNTp‐MCS‐3Flag‐T2A‐EGFP, GV571, Genechem Co., Ltd) and encoded empty vector (EV) or USP25 (AAV9‐cTnT‐USP25^oe^). The mice were injected with AAV9 via tail vein (1 × 10 E + 11 v.g./mouse) 4 weeks before I/R surgery.

### 2.2 Echocardiography

For echocardiography, the mice were anaesthetized with 3% isoflurane and placed on a plate without mechanical ventilation. The standard short‐axis section was identified to assess cardiac function in mice. Ventricular wall motion was captured using M‐Mode imaging. Echocardiographic parameters, including ejection fraction (EF) and shortening fraction (FS), were obtained using the multi‐mode small animal ultrasound imaging system (Vevo 3100, FUJIFILM Visual Sonics).

### 2.3 Assessment of ischemic area (AAR) and infarcted area size (INF)

The area at risk (AAR) is the myocardium segment vulnerable to infarction due to ischaemia, whereas the infarcted area size (INF) represents the degree of irreversible myocardial damage. After MI/RI, the LAD was re‐ligated. Then, mice abdominal cavities were incisioned and 1 mL of 2% Evans Blue staining solution (E2129, Sigma‐Aldrich) was injected via the inferior vena cava. The hearts were excised upon complete blueness, washed with PBS and stored at −20°C for 15 min. The hearts were then sliced into five sections of 1 mm thickness along the short axis. The unstained regions represent ischaemic areas, whereas blue regions indicate non‐ischaemic regions. The slices were immersed in 1% 2,3,5‐triphenyltetrazolium chloride (TTC) solution (T8170, Solarbio) for 15 min in darkness at 37°C and then fixed in 4% paraformaldehyde for 2 h at room temperature. The AAR is delineated by the non‐Evans Blue‐stained (pale plus red) area, and the INF size is quantified as the unstained (pale) portion within the AAR.

### 2.4 Serum biochemical analysis and measurement of inflammatory cytokines

Mice blood was collected and centrifuged at 3000 *g* for 15 min to obtain serum. The levels of cardiac Troponin T (cTnT, E‐EL‐M1801c, Elabscience), lactate dehydrogenase (LDH, BC0685, Solarbio), creatine kinase (CK)‐MB (Jiancheng Bioengineering Institute, Nanjing, China), cardiac natriuretic peptide (ANP, F10062) and IL‐1β (F10770) were assessed utilizing the related kit in accordance with the manufacturer's guidelines.

### 2.5 TUNEL staining and histological analysis

Frozen sections of hearts were selected for staining in accordance with the instructions of the one‐step TUNEL Apoptosis Detection Kit (C1090 for Figure 2G and C1086 for Figure 3E, Beyotime). Additionally, paraffin sections of hearts were chosen for staining, following the guidelines of the Masson staining kit (G1340, Solarbio, and E‐CK‐A322, Elabscience). We randomly selected six visual fields from each TUNEL‐stained frozen myocardial tissue sample. Subsequently, we counted the number of TUNEL‐positive cells as well as the total cell count in each visual field. The ratio of these two values was then calculated, followed by a statistical analysis.

### 2.6 Cell culture, transfection and viability assessment

The HEK‐293T human embryonic kidney cell line, NIH/3T3 mouse embryonic fibroblasts cell line and HL‐1 cell line were obtained from the Shanghai Institute of Biochemistry and Cell Biology (Shanghai, China). Neonatal rat primary cardiomyocytes (NRPCs) were isolated from the ventricles of neonatal Sprague–Dawley rats. Rats that were born within 3 days were euthanized, and their hearts were excised and washed with PBS to remove any residual blood. The heart tissue was subsequently digested using a mixture containing.8 mg/mL pancreatin (T8150, Solarbio) and.625 mg/mL collagenase (BY06021, Shanghai Boyun BioTech Co., Ltd.). Primary cardiomyocytes were then isolated from fibroblasts through the method of differential adherent culture. Finally, the myocardium cells were cultured in DMEM medium supplemented with 4.5 g/L glucose, 10% foetal bovine serum (FBS) and 5‐BrdU (HY‐15910, MedChemExpress). The cells were placed in a humidified incubator maintained at 37°C with an atmosphere of 5% CO_2_. After 48 h of culture, the cardiomyocytes exhibited regular pulsation patterns indicative of their viability for subsequent experimental applications. The HEK‐293T, NIH/3T3, HL‐1 and NRPCs were cultured in DMEM (Gibco) with 10% FBS (Vazyme) and 1% penicillin/streptomycin in a constant temperature incubator set at 37°C with 5% CO_2_.

1. In vitro cardiomyocyte pyroptosis model: Cardiomyocytes were treated with 1 µg/mL LPS (*Escherichia coli* O111:B4, Sigma‐Aldrich) for 6 h for priming, followed by stimulation with 10 µM Nigericin (Nig, HY‐100381, MedChemExpress) for 30 min to establish the cardiomyocyte pyroptosis model. LPS is solubilized in sterilized water, whereas Nigeritin is dissolved in ethanol.
2. In vitro cardiomyocyte hypoxia/reoxygenation (H/R) model: Cardiomyocytes were cultured in hypoxic conditions (1% O_2_, 5% CO_2_ and 94% N_2_ gas mixture) for 4 h with FBS and glucose‐deprived DMEM to induce hypoxic injury. Subsequently, the medium was changed to a high glucose DMEM containing FBS, and the cardiomyocytes were incubated in normoxic conditions (21% O_2_, 5% CO_2_ and 74% N_2_ gas mixture) for 6 h to induce reoxygenation injury to established a cardiomyocyte H/R model. Control groups were cultured in normoxic conditions for the corresponding times with high glucose DMEM containing FBS.
3. Plasmid transfection and USP25 gene silencing: When the cell density in the 6‐well plate reached 60%, we transfected 1 µg plasmid by using Opti‐MEM Medium (cat. no. 31985070, Thermo Fisher Scientific) containing 2 µL Lipofectamine 3000 and 2 µL P3000 (cat. no. L3000‐015, Thermo Fisher Scientific). siRNAs of 50 nM were transfected by using Opti‐MEM Medium containing 2 µL Lipofectamine 3000. The cells were transfected 24 h later for subsequent experiments. The detailed information regarding plasmids are shown in Table S1.
4. Cell viability was assessed by cell counting kit‐8 (C0038, Beyotime). Cytotoxicity was detected by an LDH release assay kit (C0016, Beyotime) according to the manufacturer's instructions.

### 2.7 Hoechst/Propidium iodide (PI) staining

Cells were fixed with 5 µL PI stain (CA1120, Solarbio) and incubated at 4°C for a duration of 30 min. Cells were counterstained with Hoechst to stain nuclei. Microscopic visual fields were captured randomly using an inverted fluorescence microscope, and the pertinent experimental data were meticulously gathered.

### 2.8 ASC oligomerization and fluorescence microscopy

To initiate ASC oligomerization, cells underwent treatment and were subsequently lysed by using NP‐40 lysis buffer (P0013F) sourced from Beyotime and agitated on a shaker for 15 min at 4°C before subjecting to centrifugation at 6000 *g* for 15 min. The resulting cell pellets underwent cross‐linking with newly prepared disuccinimidyl suberate (2 mM) for 30 min at 37°C and were then collected through additional centrifugation. The pellets were then dissolved in 5X SDS sample buffer and analysed via using western blot.

ASC specks were performed by using fluorescence microscopic analysis. Cells were fixed with 4% paraformaldehyde and permeabilized with.1% Triton X‐100. Nonspecific binding sites were blocked by 5% bovine serum albumin and then incubated with ASC antibody (67824S, 1:200, CST) overnight at 4°C. Alexa Fluor 594–labelled (33112ES60, LABSELECT, 1:200) secondary antibody was applied for detection. Cells were counterstained with DAPI to stain nuclei. Microscopic visual fields were captured randomly by a blinded experimenter using laser confocal microscopy (spin SR, OLYMPUS).

### 2.9 Human heart samples

Human myocardial samples were collected as previously described.^20^

### 2.10 Transcriptome sequencing

In compliance with the manufacturer's guidelines, TRIzol reagent (cat. no. 15596018, Invitrogen) was used to extract total RNA from heart tissue. Library construction and transcriptome sequencing were performed on an Illumina Novaseq 6000 (LC‐Bio Technology Co., Ltd.) following the vendor's recommendations.

### 2.11 Real‐time quantitative polymerase chain reaction (RT‐qPCR)

Total RNA from human myocardial samples, mouse heart tissues and culture cells was isolated and purified by using TRIzol reagent. Then RNA was reverse‐transcribed to cDNA using PrimeScript RT reagent Kit (cat. no. DRR037A, Takara). Quantitative polymerase chain reaction (PCR) was performed by using SYBR Green reagent kits (cat. no. DRR037A, Takara) under the following conditions: 95°C for 10 min, followed by 40 cycles of 95°C for 15 s, and 60°C for 60 s. Target mRNA levels were normalized to β‐actin. Primers were purchased from Sangon Biotech as shown in Table S2.

### 2.12 Co‐immunoprecipitation (Co‐IP) and western blot analysis

For co‐immunoprecipitation (Co‐IP), proteins were extracted from animal myocardial tissue (10 mg) and primary cardiomyocyte (1 × 10^7^) samples with.4 mL RIPA buffer. Then, the protein lysates were incubated with primary antibody (overnight, 4°C), and part of the lysate was retained as an input sample. After that, the protein lysates were precipitated with protein G‐Sepharose beads (6–12 h, 4°C). After washed by PBS, protein G‐Sepharose beads were prepared for western blot.

Proteins from animal myocardial tissues and cell samples were extracted with RIPA buffer (P0013C, Beyotime Biotechnology). The procedures for western blot were implemented as previously described.^19^ Primary antibodies against ASC (67824S, 1:1000, CST), Caspase‐1 (sc‐56036, 1:200, Santa Cruz), Ub (sc‐8017, 1:200, Santa Cruz), β‐actin (AC026, 1:1000, Abbkine), NLRP3 (ag‐20B‐0014‐C100, 1:1000, Adipogen), GSDMD (ab215203, 1:1000, Abcam), USP25 (ab187156, 1:1000, Abcam), and IL‐1β (ab9722,1:1000, Abcam), COL‐1 (14695‐1‐AP, 1:1000, Proteintech), FLAG (20543‐1‐AP, Proteintech), GAPDH (60004‐1‐Ig, 1:1000, Proteintech), TGF‐β (21898‐1‐AP, 1:1000, Proteintech), HA (M20003S, 1:1000, Abmart), p‐P65 (3033, 1:1000, CST), P65 (8242, 1:1000, CST), P38 (8690, 1:1000, CST), p‐P38 (463190, 1:1000, CST), JNK (9252, 1:1000, CST), p‐JNK (4668, 1:1000, CST), ERK (4695, 1:1000, CST, p‐ERK (4370, 1:1000, CST) were used. Horseradish peroxidase–conjugated goat anti‐rabbit secondary antibody (1:2000, A0208, Beyotime) and goat anti‐mouse secondary antibody (1:2000, A0216, Beyotime) were employed.

### 2.13 Co‐IP combined with LC–MS/MS analysis

NRPCs were transfected with Flag‐USP25 plasmids before being subjected to H/R treatment. Anti‐Flag and protein G‐Sepharose beads were added to the cell samples for Co‐IP. The binding proteins were extracted from Co‐IP beads using SDT lysis buffer. Protein was digested to peptide via the FASP method. Then, the LC–MS/MS analysis was carried out by PTM Bio Co., Ltd.

### 2.14 NLRP3 ubiquitination site analysis

HL‐1 were transfected with EV or Flag‐USP25 plasmids before being subjected to H/R treatment. The protein is extracted from HL‐1 and NLRP3 antibody was used for NLRP3 enrichment. The LC–MS/MS analysis and the subsequent ubiquitylome analysis were carried out by BIOPROFILE.

### 2.15 Statistical analysis

The results in this study are presented as mean ± standard deviation. In vitro data represent the mean of multiple cultured cells, and normal distribution was inferred based on the central limit theorem. In vivo data sample sizes were all ≥6. The normal distribution of data points was assessed using the Shapiro–Wilk test, where a *p* value >.05 indicated approximate conformity to a normal distribution. Student's *t*‐test was utilized for comparisons between two groups, whereas one‐way ANOVA followed by Tukey's post hoc test was employed for comparisons among multiple groups. All data analyses were conducted using GraphPad Prism 8.0 software (GraphPad).

## 3 RESULTS

### 3.1 USP25 is down‐regulated in MI/RI myocardium tissues

**FIGURE 1:**
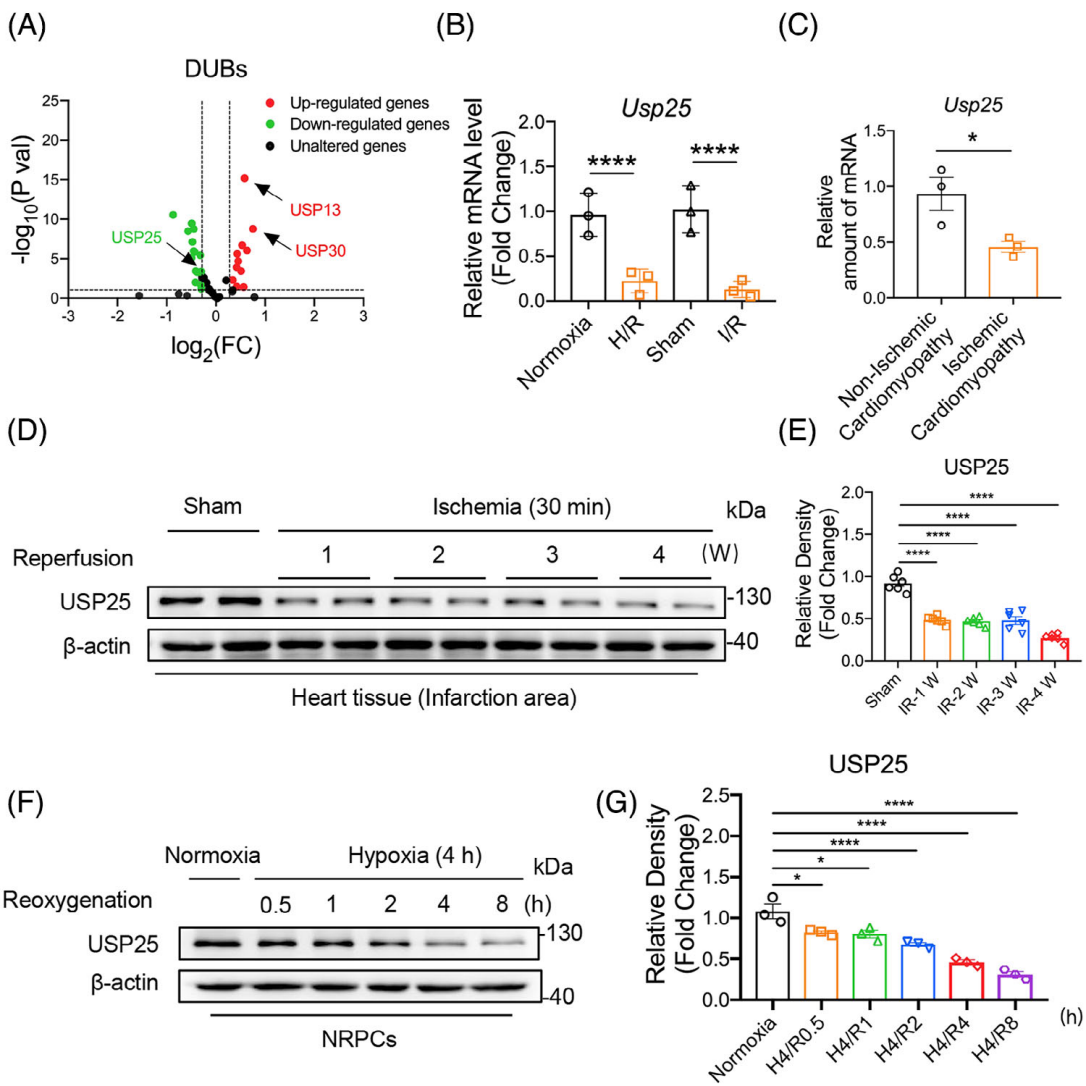
Ubiquitin‐specific protease 25 (USP25) is down‐regulated in myocardial ischemia/reperfusion injury (MI/RI) myocardium tissues. (A) RNA transcriptome sequencing was used to detect mRNA expression of UPS family members of deubiquitinating enzymes (DUBs) during MI/RI. RNA transcriptome sequencing was performed on Sham (*n* = 3) and I/R‐induced (*n* = 3) mice heart samples, respectively. We use log_2_ of the fold change as the source of data for the *x*‐axis and −log_10_ of the *p* value as the source of data for the *y*‐axis. Fold change > 1.5 times and *p* ˂ .05 indicate statistically significant. Myocardial tissue in mice was subjected to 30 min of ischaemia, followed by a subsequent period of 4 h of reperfusion to induce I/R injury. Neonatal rat primary cardiomyocytes (NRPCs) were cultured in glucose‐ and foetal bovine serum (FBS)‐deprived DMEM under hypoxic conditions for a duration of 4 h to elicit hypoxic injury. Subsequently, the medium was replaced with high‐glucose media containing 10% FBS, after which the cardiomyocytes were incubated under normoxic conditions for an additional 6 h to induce hypoxia/reoxygenation (H/R) injury, compared to the sham group. (B) Myocardial tissue in mice was subjected to 30 min of ischaemia, followed by a subsequent period of 4 h of reperfusion to induce I/R injury. NRPCs were cultured in glucose‐ and FBS‐deprived DMEM under hypoxic conditions for a duration of 4 h to elicit hypoxic injury. Subsequently, the medium was replaced with high‐glucose media containing 10% FBS, after which the cardiomyocytes were incubated under normoxic conditions for an additional 6 h to induce H/R injury. Real‐time quantitative polymerase chain reaction (RT‐qPCR) analysis of the USP25 mRNA expression that was differentially expressed in RNA transcriptome sequencing induced by cardiomyocyte H/R injury and myocardial I/R injury (*n* = 3). (C) RT‐qPCR analysis of the mRNA expression of USP25 in myocardial tissues of ischaemic cardiomyopathy patients and normal heart tissues (*n* = 3). (D and E) Representative western blot (D) and densitometric quantification (E) for USP25 in I/R‐induced heart tissue in the area of myocardial injury at different reperfusion time points and normal heart tissues in mice (*n* = 6). (F and G) Representative western blot (F) and densitometric quantification (G) for USP25 in H/R‐induced NRPCs at different reoxygenation time points and normoxia‐treated NRPCs (*n* = 3). Significance is defined as *p < .05, **p < .01, ****p < .0001.

High‐throughput transcriptome sequencing was performed to screen the expression profile of USP family member genes in the heart tissues of the sham operation (Sham) mice and the myocardial I/R mice. The RNA‐seq data showed different expression changes of some USP family genes in I/R mouse hearts (Figure 1A). We selected the top 30 up‐ and down‐regulated DUBs for verification using real‐time qPCR assay in both murine myocardial tissues and cultured cardiomyocytes, respectively. Interestingly, the results showed that only USP25 mRNA level was significantly decreased in both I/R‐induced myocardium tissues and H/R‐induced cardiomyocytes (Figure 1B and Figure S1A,B). We also confirmed that the mRNA level of USP25 in the myocardial tissue of patients with ischaemic cardiomyopathy was significantly lower than that in patients with non‐ischaemic cardiomyopathy (Figure 1C). In addition, our results also showed that the protein level of USP25 was decreased in a time‐dependent manner in both I/R‐induced myocardium tissues (Figure 1D,E) and H/R‐induced cardiomyocytes (Figure 1F,G). This changing profile of USP25 indicated that USP25 may play a role in MI/RI.

### 3.2 USP25 deficiency exacerbates MI/RI in mice

**FIGURE 2:**
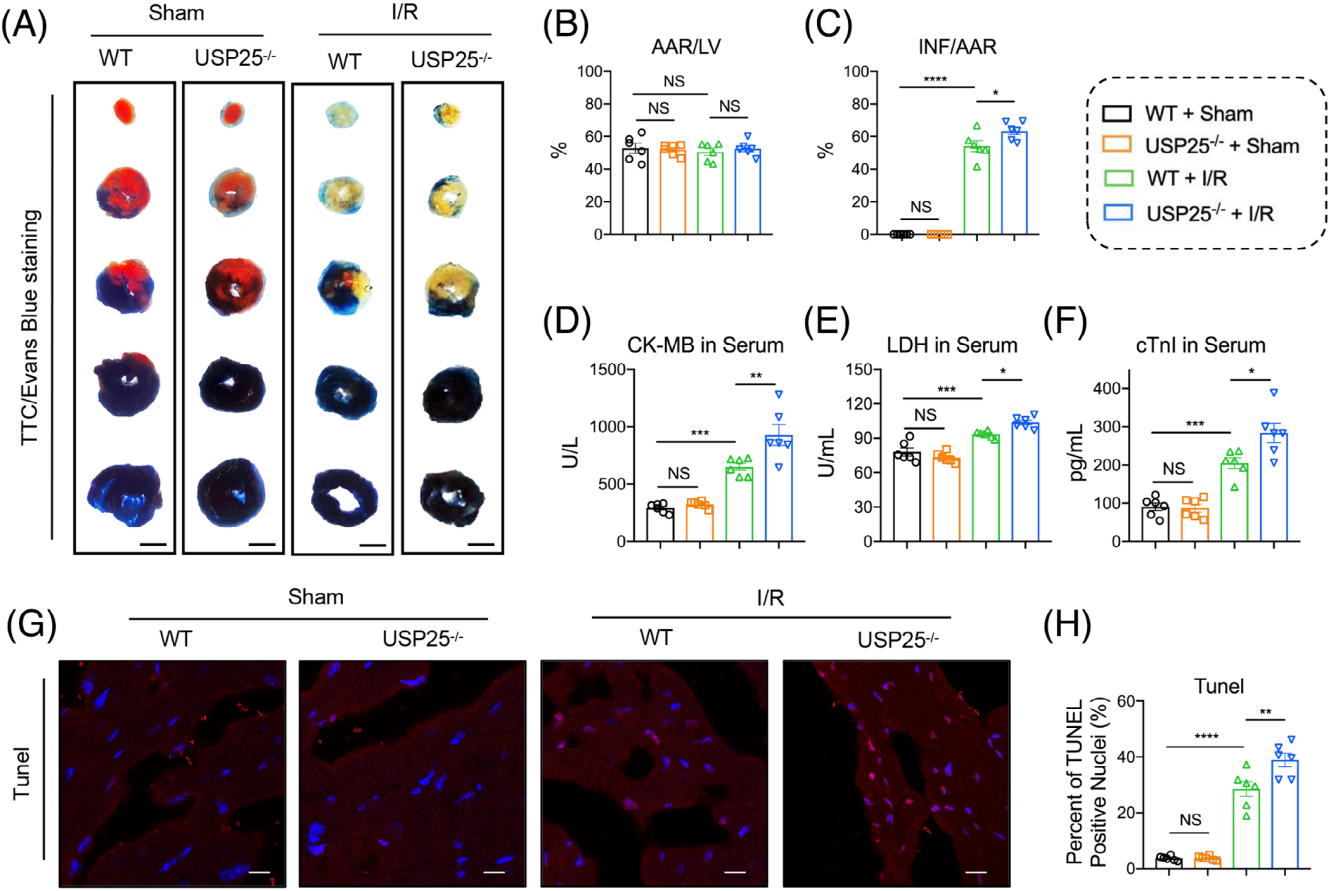
Ubiquitin‐specific protease 25 (USP25) deficiency exacerbates myocardial ischemia/reperfusion injury (MI/RI) in mice. The whole‐body USP25 knockout (USP25^−/−^) mice and wild‐type littermates were subject to acute MI/RI (30 min ischemia followed by 4 h reperfusion) and sham operation. (A–C) Representative images of sections stained with Evans Blue and 2,3,5‐triphenyltetrazolium chloride (TTC) (A) and quantitative data for area at risk (AAR) and left ventricular (LV) area (B) and infarcted area size (INF) and AAR (C). (D–F) The content of creatine kinase (CK)‐MB (D), lactate dehydrogenase (LDH) (E) and cTnI (F) in serum from each group. (G and H) Representative images of sections stained with TUNEL in the infarcted area (red: positive cells; scale bar, 1 mm and 100 µm; G) and quantitative data (H). Significance is defined as *p < .05, **p < .01, ***p < .001, ****p < .0001. The abbreviation ‘NS’ denotes no statistical significance (*p* > .05).

The whole‐body USP25 knockout (USP25^−/−^) mice and WT littermates were subjected to an acute cardiac I/R model with 30 min ischaemia followed by 4 h of reperfusion or sham operation. TTC/Evan Blue staining found that USP25 deficiency led to a more severe myocardial infarct area induced by I/R (Figure 2A–C). Cardiac injury markers, including serum CK‐MB, LDH, and cTnI levels, showed similar changing trends (Figure 2D–F). Moreover, the TUNEL‐positive cells in myocardium tissues were increased when mice were under myocardial I/R treatment and were further aggravated in USP25^−/−^ mice (Figure 2G,H).

### 3.3 USP25 deficiency aggravates I/R‐induced cardiac remodelling

**FIGURE 3:**
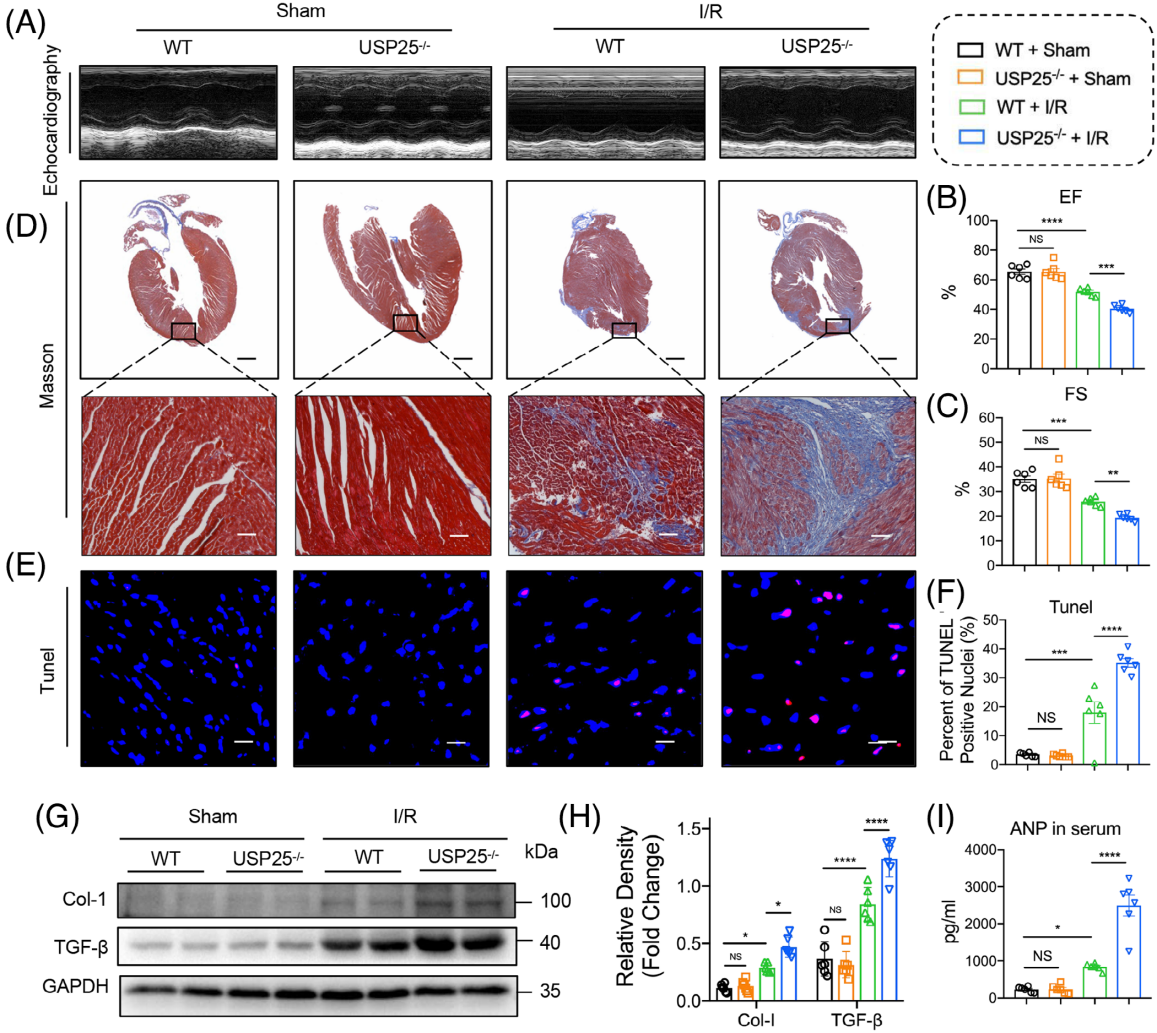
Ubiquitin‐specific protease 25 (USP25) deficiency aggravates ischemia/reperfusion (I/R)‐induced cardiac remodelling. The whole‐body USP25 knockout (USP25^−/−^) mice and wild‐type littermates were subject to chronic myocardial I/R injury (MI/RI) (30 min ischaemia followed by 2‐week reperfusion) and sham operation. (A) Representative M‐mode echocardiographic images from each group in mice. (B and C) Ejection fraction (EF, B) and fractional shortening (FS, C). (D) Representative images of Masson staining of heart sections (scale bar, 1 mm and 50 µm). (E and F) Representative images of sections stained with TUNEL in the infarcted area (green: positive cells; scale bar, 100 µm; E) and quantitative data (F). (G and H) Representative western blot results of Col‐1 and TGF‐β in heart tissues (G) and quantitative data (H). (I) The content of ANP in serum from each group. Significance is defined as *p < .05, **p < .01, ***p < .001, ****p < .0001. The abbreviation ‘NS’ denotes no statistical significance (*p* > .05).

Myocardial I/R injury further causes ventricular remodelling and heart failure if treatment is not prompt. We therefore assessed whether USP25 deficiency could aggravate chronic I/R injury (30 min ischaemia followed by 2‐week reperfusion) and concomitant heart failure. As shown by non‐invasive echocardiography, USP25 deficiency developed more severe cardiac dysfunction in MI/RI‐induced mice, as evidenced by the decreased EF and FS (Figure 3A–C). Consistently, the degree of interstitial fibrosis in heart tissues was significantly increased in MI/RI‐induced USP25^−/−^ mice than in WT mice (Figure 3D). USP25^−/−^ mice also had an obviously increased TUNEL‐positive cell in myocardium tissues compared with WT mice challenged by MI/RI (Figure 3E,F). In addition, USP25 deficiency aggravated the fibrotic markers (COL‐I and TGF‐β) in MI/RI‐induced mouse heart tissues (Figure 3G,H). Serum ANP levels followed similar trends (Figure 3I). Together, these results show that USP25 deficiency exacerbates myocardial I/R‐changed remodelling and dysfunction.

### 3.4 Identification of NLRP3 as the potential substrate protein of USP25 in I/R‐induced cardiomyocyte injury

**FIGURE 4:**
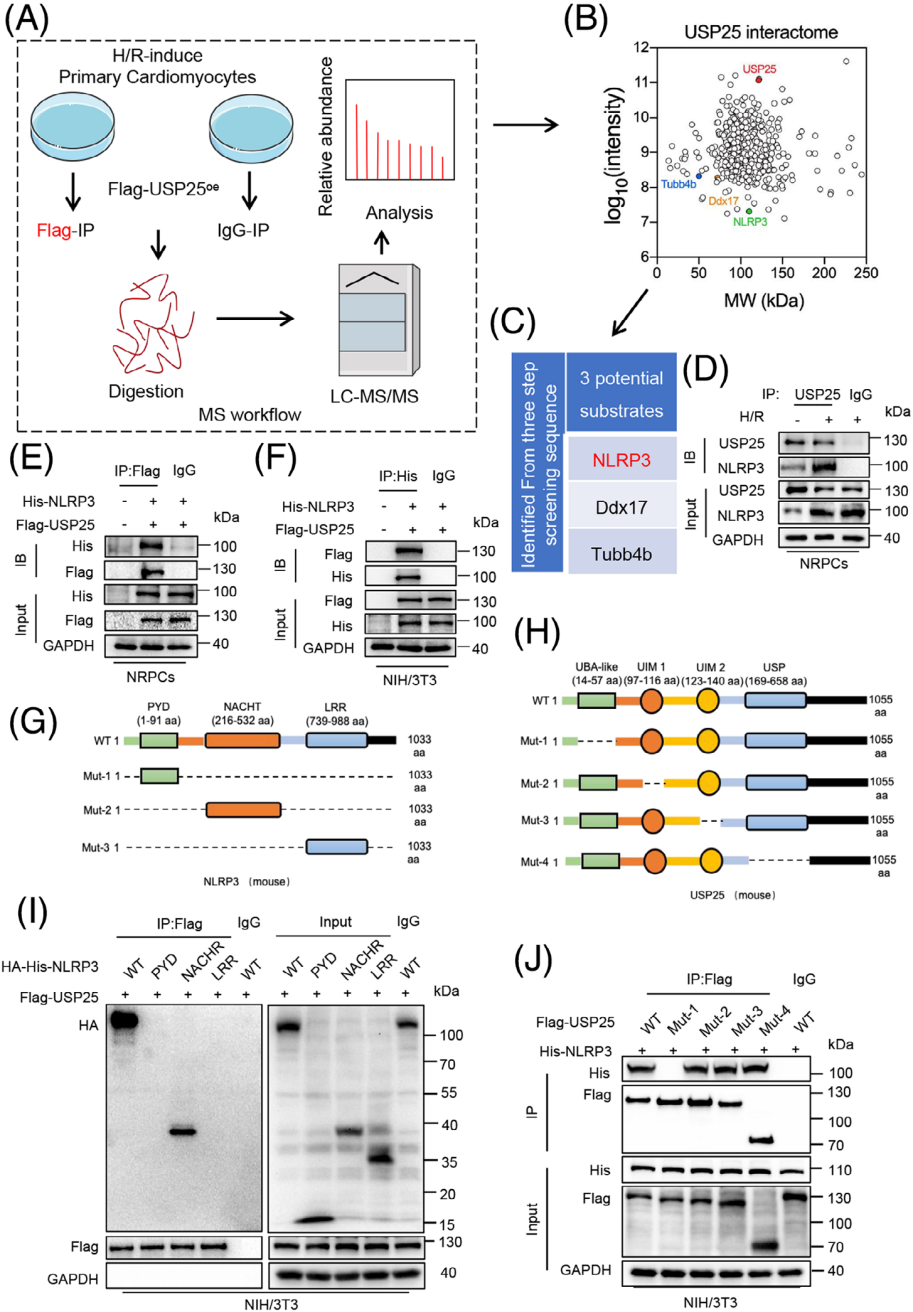
Identification of NLRP3 as the potential substrate protein of ubiquitin‐specific protease 25 (USP25) in ischemia/reperfusion (I/R)‐induced cardiomyocyte injury. (A) The workflow of USP25 substrate screening. Neonatal rat primary cardiomyocytes (NRPCs) were transfected with Flag‐USP25 plasmids, followed by hypoxia/reoxygenation (H/R) stimulation. Anti‐Flag and protein G‐Sepharose beads were added to the cell samples for coimmunoprecipitation (Co‐IP). The binding proteins were extracted, digested to peptide, and then performed for LC–MS/MS analysis. (B) Two‐dimensional (2D) plot with log_10_ signal intensity of the quantified proteins on the *y* axis (revealing the enrichment in Flag‐USP25‐IP) and molecular weight (MW) of protein on the *x* axis. (C) The table shows the candidate substrates of USP25 screened by the LC–MS/MS. (D and E) Co‐IP of endogenous (D) and exogenous (E) USP25 and NLRP3 in primary cardiomyocytes treated with or without H/R injury. Endogenous USP25 was immunoprecipitated by anti‐USP25 antibody. Exogenous USP25 was immunoprecipitated by anti‐Flag antibody. IgG, immunoglobulin G. (F) Co‐IP of USP25 and NLRP3 in NIH/3T3 cells co‐transfected with Flag‐USP25 and His‐NLRP3 plasmids. Exogenous NLRP3 was immunoprecipitated by anti‐His antibody. (G) Schematic illustration of the NLRP3 domain deletion construct used in Figure 5I. (H) Schematic illustration of the USP25 domain deletion construct used in Figure 5J. (I) Co‐IP of wt‐NLRP3, mut‐NLRP3 and USP25 in NIH/3T3 cells co‐transfected with overexpression plasmids of HA‐His‐wt‐NLRP3, HA‐His‐mut‐NLRP3 and Flag‐USP25. Exogenous normal or mutated NLRP3 was immunoprecipitated by anti‐Flag antibody. (J) Co‐IP of wt‐USP25, mut‐USP25 and NLRP3 in NIH/3T3 cells co‐transfected with overexpression plasmids of Flag‐wt‐USP25, Flag‐mut‐USP25 and His‐NLRP3. Exogenous normal or mutated USP25 was immunoprecipitated by anti‐Flag antibody. IgG, immunoglobulin G.

DUBs perform their functions by modifying their substrate proteins. In order to identify the substrate protein regulated by UPS25 in MI/RI, we transfected USP25 plasmid into NRPCs for 24 h before challenging it with H/R. Then, Co‐IP combined with liquid chromatography–tandem mass spectrometry (LC–MS/MS) was applied to explore potential substrate proteins binding to USP25 (Figure 4A). Among a total of 432 proteins (score > 50) identified by LC–MS/MS, 162 substrate proteins with >2‐fold change were selected as candidate proteins (Figure 4B). Due to a certain degree of false positives in the results of LC–MS/MS, we repeated the experiment with the same conditions and identified three proteins, Ddx17, Tubb4b and NLRP3, as USP25‐binding substrates in the outcomes of two LC–MS/MS experiments. Compared with Ddx17 and Tubb4b, numerous studies have reported that cardiomyocyte NLRP3 plays a significant role in the pathogenesis and development of MI/RI.^21^, ^22^ MI/RI contributes to mitochondrial dysfunction and the generation of reactive oxygen species,^23^ which activate the NLRP3 inflammasome through modulation of nuclear factor‐κB.^24^ It is important to note that USP25 has been identified as playing a crucial role in the regulation of IL‐1β production by NLRP3.^25^ In addition, cardiomyocyte‐derived NLRP3 and its associated pyroptosis exacerbate MI/RI.^26^ Conversely, the pharmacological inhibition of NLRP3 in myocytes has been shown to alleviate MI/RI,^27^ indicating that NLRP3 in myocytes plays a crucial role in this pathological process. Therefore, it is very likely that USP25 negatively regulates MI/RI through targeting NLRP3 in cardiomyocytes. Hence, we selected NLRP3 as a potential target protein of USP25 in cardiomyocytes for further investigations. As shown in Figure 4D, we discovered that the interaction of USP25 and NLRP3 in NRPCs was significantly increased under H/R stimulation. Subsequently, USP25 and NLRP3 plasmids were co‐transfected into NRPCs and NIH/3T3 cells, confirming the combination of USP25 and NLRP3 (Figure 4E,F). Structurally, NLRP3 is consisted of a central nucleotide oligomerization domain (defined as NACHT), a C‐terminal leucine‐rich repeat (LRR) domain and an n‐terminal effect domain (pyrin domain or PYD) interacting with downstream signalling molecules,^28^ and USP25 contains four structural segments, namely, the ubiquitin‐specific enzymatic solution domain (USP), the ubiquitin‐associated domain (UBA) and two tandem ubiquitin interaction moieties (UIM) (Figure 4G,H). We constructed USP25 and NLRP3 plasmids mutated in different regions and performed Co‐IP experiments. The results revealed that USP25 binds to the NACHT domain of NLRP3 through its UBA domain (Figure 4I,J).

### 3.5 USP25 negatively regulates NLRP3 activity both in vitro and in vivo

**FIGURE 5:**
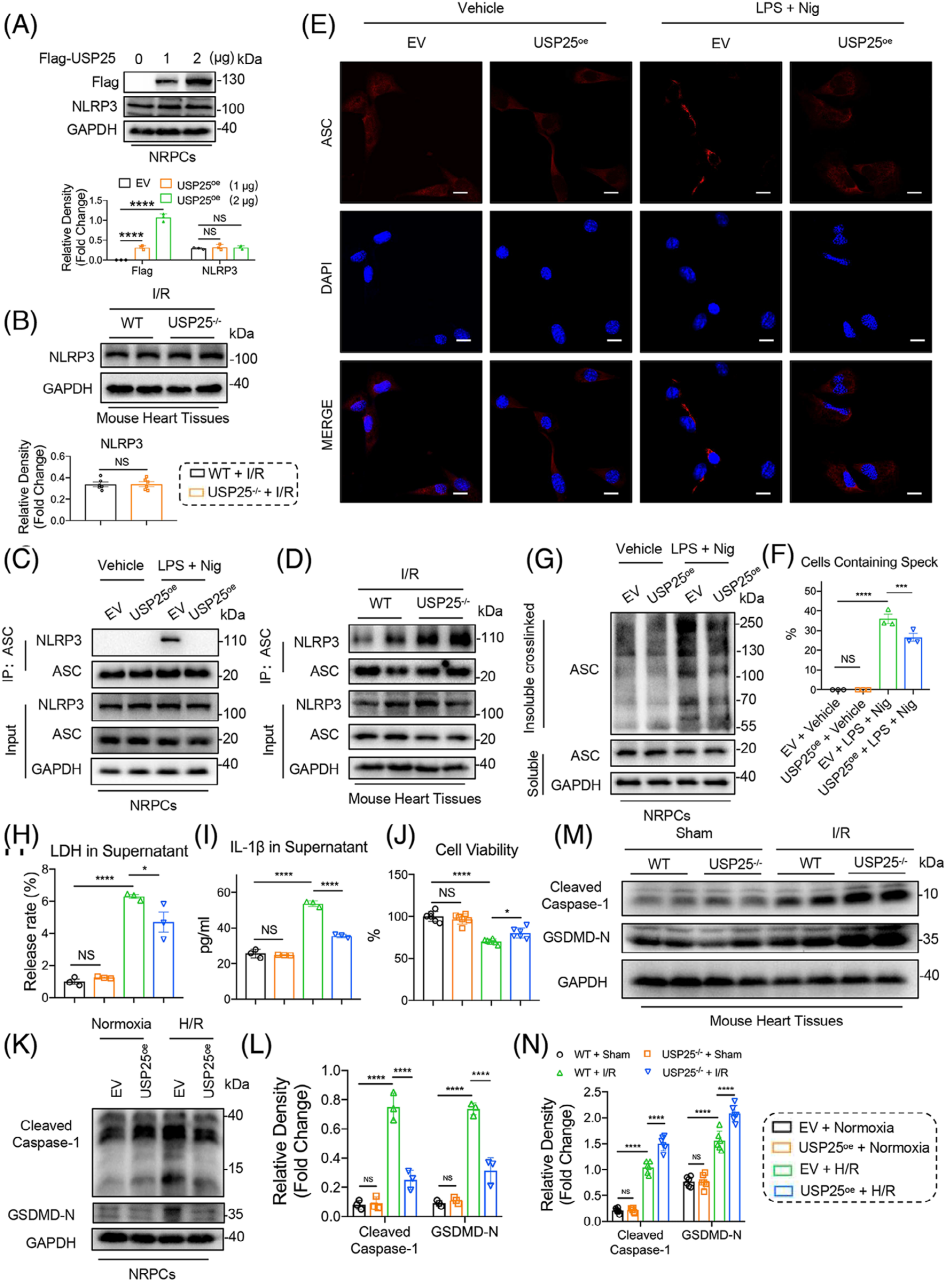
Ubiquitin‐specific protease 25 (USP25) negatively regulates NLRP3 activity both in vitro and in vivo. (A and B) Representative western blot for USP25 and NLRP3 in neonatal rat primary cardiomyocytes (NRPCs) transfected with overexpression plasmids of Flag‐USP25 (A, *n* = 3) and ischaemia/reperfusion (I/R)‐induced heart tissues from wild‐type mice and USP25^−/−^ mice (B, *n* = 6). (C and D) Co‐immunoprecipitation of endogenous ASC and NLRP3 in NRPCs treated with or without LPS/Nig stimulation (C) and I/R‐induced heart tissues from wild‐type mice and USP25^−/−^ mice (D). Endogenous ASC was immunoprecipitated by using anti‐ASC antibody. NRPCs were subject to 1 µg/mL LPS for 6 h for priming, followed by stimulating with 10 µM Nigericin (Nig) for 30 min after transfection with plasmids. Wild‐type mice and USP25^−/−^ mice were subject to 30 min ischaemia followed by 4 h reperfusion. (E and F) Immunofluorescence staining of ASC in NRPCs. ASC specks were detected by immunostaining using anti‐ASC antibody, and cells were counter‐stained with nuclei‐staining DAPI (E). The histogram quantitates the percentage of cells that exhibit ASC speck formation (F) (scale bar, 20 µm). (G) Co‐immunoprecipitation of ASC in chemically cross‐linked NP‐40 insoluble fractions and in NP‐40‐soluble fractions from cell lysates of NRPCs. (H and I) The release of lactate dehydrogenase (LDH) (H) and IL‐1β (I) in culture medium. NRPCs were subject to hypoxia/reoxygenation (H/R) injury (4 h hypoxia followed by 6 h reoxygenation) after transfection with plasmids. (J) Cell viability of NRPCs. (K and L) Representative western blot results of Cleaved Caspase‐1 and gasdermin D (GSDMD)‐N in NRPCs (K) and quantitative data (L). (M and N) Representative western blot results of Cleaved Caspase‐1 and GSDMD‐N in mouse myocardial tissues (M) and quantitative data (N). Significance is defined as *p < .05, **p < .01, ****p < .0001. The abbreviation ‘NS’ denotes no statistical significance (*p* > .05).

We next aimed to explore how USP25 regulates NLRP3 pathway. DUBs usually regulate the stability or function of the substrate protein by removing its ubiquitin. As shown in Figure 5A,B, either USP25 overexpression or knockout did not affect the stability of NLPR3 in NRPCs and mouse heart tissues under I/R treatment (Figure 5A,B). Therefore, we speculate that USP25 may regulate the activity of NLRP3. NLRP3–ASC interaction and ASC oligomerization are the key steps in the activation of the NLRP3 inflammasomes. We found that USP25 overexpression significantly reduced the binding of NLRP3 to ASC induced by LPS/nigericin in (Figure 5C). Similarly, USP25 knockout increases NLRP3 binding to ASC in I/R‐induced mouse heart tissues (Figure 5D). Immunofluorescence assay showed that LPS/Nigericin treatment significantly increased ASC oligomerization as evidenced by increased ASC spots, whereas the overexpression of USP25 inhibited this change in NRPCs (Figure 5E,F). We also treated the cell lysates with a bifunctional chemical cross‐linker and performed ASC immunoblotting to evaluate the ASC oligomerization. As expected, LPS/Nigericin‐induced oligomerization of ASC was significantly reduced by USP25 overexpression in NRPCs (Figure 5G). However, a possible substrate for USP25 could be ASC and not necessarily NLRP3. Thus, we conducted Co‐IP experiments and confirmed that there is no direct binding between USP25 and ASC (Figure S2).

These data show that USP25 negatively regulates NLRP3 activity but does not affect the protein stability of NLRP3.

Activation of NLRP3 inflammasome induces pyroptosis in cardiomyocytes, which promotes I/R‐induced cardiac injury. We then examined if USP25 down‐regulates H/R‐induced cardiomyocyte pyroptosis. We showed that the overexpression of USP25 in NRPCs (Figure S3A,B) inhibited the H/R‐increased levels of LDH and IL‐1β (Figure 5H,I). USP25 also increased the cell viability of H/R‐challenged NRPCs (Figure 5J) and significantly attenuated H/R‐induced gasdermin D (GSDMD) activation as shown by the reduced cleavage of GSDMD and Caspase‐1 (Figure 5K,L). Then, silencing USP25 by using siRNA (Figure S4A) further increases the release of LDH and IL‐1β in H/R‐challenged NRPCs (Figure S4B,C). Inhibition of USP25 expression in cardiomyocytes under H/R treatment led to a further decrease in cell viability (Figure S4D), accompanied by the increased protein level of cleaved Caspase‐1 and GSDMD N‐terminal fragment (GSDMD‐N) (Figure S4E,F). Furthermore, the PI content was decreased by UPS25 overexpression when NRPCs were subjected to H/R (Figure S5A) and increased when USP25 was knocked down by siRNA (Figure S5B). Consistently, protein levels of cleaved Caspase‐1 and GSDMD‐N were significantly increased in the mouse heart tissues undergoing myocardial I/R stimulation and were further increased by USP25 knockout (Figure 5M,N). These data show that USP25 negatively regulates NLRP3 activity and cardiomyocyte pyroptosis both in vitro and in vivo.

### 3.6 USP25 deubiquitinates NLRP3 via its active site C178

**FIGURE 6:**
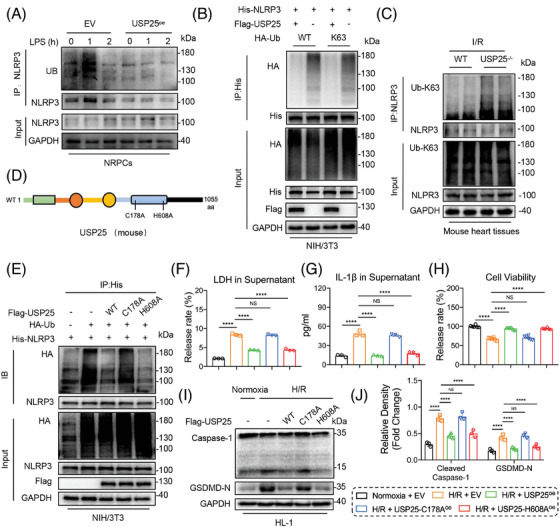
Ubiquitin‐specific protease 25 (USP25) deubiquitinates NLRP3 via its active site C178. (A) Immunoprecipitation of NLRP3 in neonatal rat primary cardiomyocytes (NRPCs) that co‐transfected with overexpression plasmids of empty vector (EV) or USP25 overexpression plasmid (USP25^oe^) and then subjected to LPS (1 µg/mL) pulse‐chase assay. Ubiquitinated NLRP3 was detected by immunoblotting using an UB antibody to clarify the ubiquitination pattern of NLRP3 regulated by USP25. (B) Immunoprecipitation of NLRP3 in NIH/3T3 cells that co‐transfected with overexpression plasmids of His‐NLRP3, HA‐Ub and HA‐Ub‐K63 and then subjected to MG132 (10 µM). Ubiquitinated NLRP3 was detected by immunoblotting using a His‐specific antibody to clarify the ubiquitination pattern of NLRP3 regulated by USP25. (C) Immunoprecipitation of NLRP3 in ischemia/reperfusion (I/R)‐induced heart tissues from wild‐type mice and USP25^−/−^ mice. Ubiquitinated NLRP3 was detected by immunoblotting using an Ub‐K63 antibody to clarify the K63 ubiquitination level of NLRP3 regulated by USP25. (D) Schematic illustration of the USP25 active site deletion construct used in parts (E)–(J). (E) Immunoprecipitation of NLRP3 in NIH/3T3 cells that co‐transfected with overexpression plasmids of wt‐Flag‐USP25, mut‐Flag‐USP25, His‐NLRP3 and HA‐Ub and then subjected to MG132 (10 µM). Ubiquitinated NLRP3 was detected by immunoblotting using a His‐specific antibody to clarify the ubiquitination level of NLRP3 regulated by the active site of USP25. (F and G) The release of lactate dehydrogenase (LDH) (F) and IL‐1β (G) in HL‐1. (H) Cell viability of HL‐1. (I and J) Representative western blot results of Cleaved Caspase‐1 and gasdermin D (GSDMD)‐N in HL‐1 (I) and quantitative data (J). Significance is defined as ****p < .0001. The abbreviation ‘NS’ denotes no statistical significance (*p* > .05).

We were interested in the regulatory mechanisms of USP25 on NLRP3 activity. We thus treated WT and USP25‐overexpressed NRPCs with LPS for various times and analysed the ubiquitination status of NRRP3. LPS promoted time‐dependent polyubiquitination of NLRP3 in WT cells, and this was considerably reduced in USP25‐overexpressed NRPCs, indicating that USP25 removes LPS‐induced ubiquitination of NLRP3 (Figure 6A). We next defined the type of linkages in the polyubiquitin chains of NLRP3 that are removed by USP25. It was found that ubiquitin molecules on NLRP3 were significantly decreased in USP25‐overexpressed cells transfected with Ub‐WT or Ub‐K63 plasmid compared to WT cells (Figure 6B). We also detected that the K63 ubiquitination level of NLRP3 was significantly increased in myocardial tissues with I/R injury from USP25 knockout mice (Figure 6C). These data demonstrate that USP25 removes K63‐linked ubiquitination of NLRP3 protein.

The cysteine at 178 site (C178) and the histidine at 608 site (H608) have been reported as catalytic motifs responsible for the deubiquitinating function of USP25.^19^, ^29^ Therefore, we constructed mutant USP25 plasmids with C178A (mutation of cysteine to alanine at C178) or H608A (mutation of histidine to alanine at H608) (Figure 6D). The ability of USP25^C178A^, but not USP25^H608A^, to remove ubiquitin molecules from NLRP3 was significantly decreased (Figure 6E), although both USP25^C178A^ and USP25^H608A^ still bind to NLRP3 (Figure S6). We also observed that USP25^C178A^ failed to reduce the H/R‐induced release of LDH and IL‐1β, compared with USP25^WT^ (Figure 6F,G). USP25^C178A^ also lost its effect on increasing the cell viability of NRPCs (Figure 6H) and attenuating H/R‐induced NLRP3 inflammasome activation in NRPCs (Figure 6I,J). In contrast, USP25^H608A^ keeps the similar ability with USP25^WT^ (Figure 6E–J). These results demonstrate that USP25 deubiquitinates NLRP3 in K63‐linked manner via its active site C178 and then inhibits NLRP3 activation in NRPCs.

### 3.7 USP25 removes ubiquitin molecules from K243 residue of NLRP3

**FIGURE 7:**
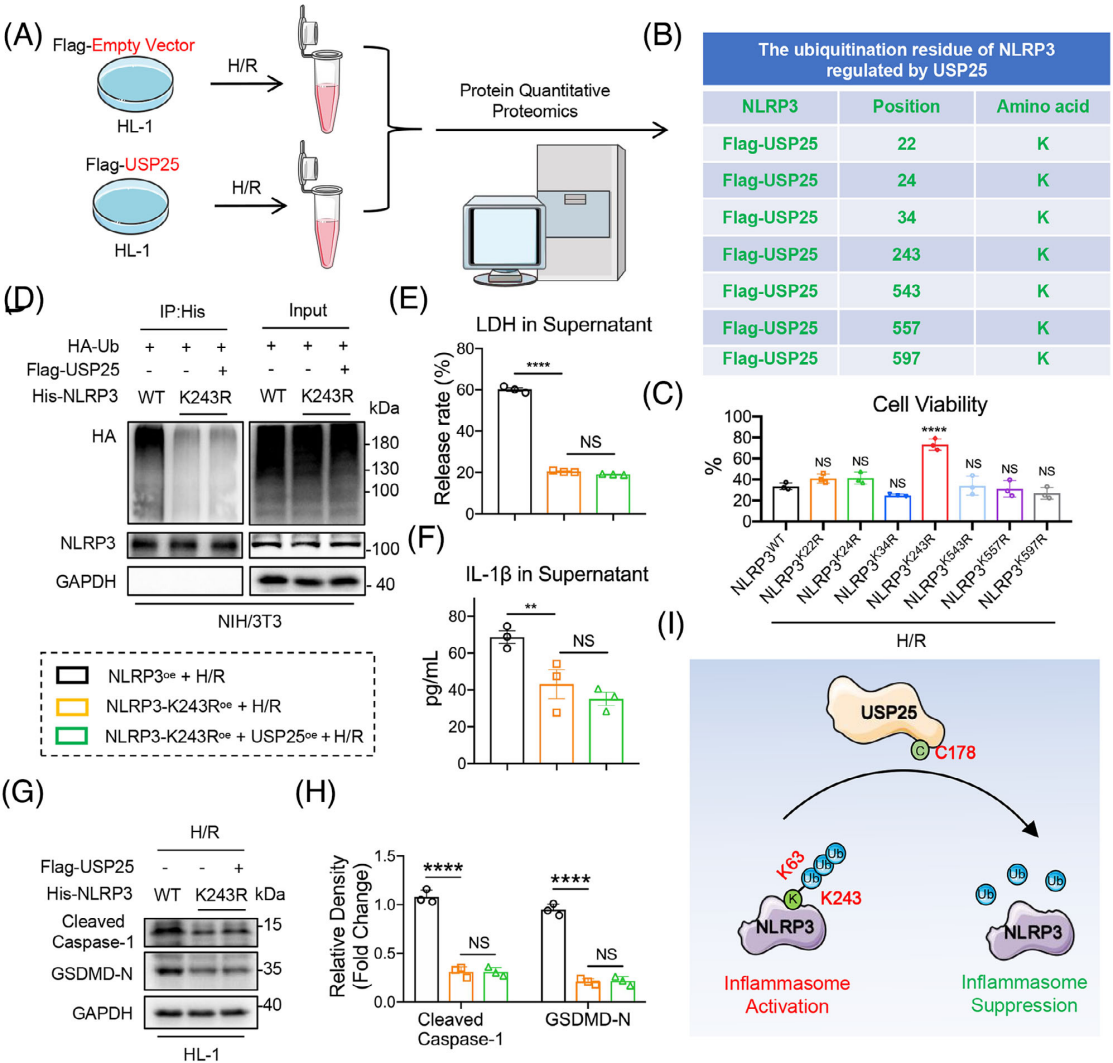
Ubiquitin‐specific protease 25 (USP25) removes ubiquitin molecules from K243 residue of NLRP3. HL‐1 cells were subject to hypoxia/reoxygenation (H/R) injury (4 h hypoxia followed by 6 h reoxygenation) after transfection with plasmids. (A) Schematic illustration of a coimmunoprecipitation (Co‐IP)‐based ubiquitinated peptide enrichment analysis for screening ubiquitination lysine residues on NLRP3 regulated by USP25. (B) The table shows that ubiquitination lysine residues on NLRP3 were identified from ubiquitinome analysis. (C) Cell viability of HL‐1. NS, represents *p* > .05 versus NLRP3^WT^ + H/R; ****p < .0001 versus NLRP3^WT^ + H/R. (D) Immunoprecipitation of NLRP3 in NIH/3T3 cells that co‐transfected with overexpression plasmids of wt‐His‐NLRP3 (NLRP3^WT^), mut‐His‐NLRP3 (NLRP3^K243R^), Flag‐USP25 and HA‐Ub and then subjected to MG132 (10 µM). Ubiquitinated NLRP3 was detected by immunoblotting using a His‐specific antibody to clarify the ubiquitination lysine residues of NLRP3‐regulated USP25. (E and F) The release of lactate dehydrogenase (LDH) (E) and IL‐1β (F) in HL‐1. (G and H) Representative western blot results of Cleaved Caspase‐1 and gasdermin D (GSDMD)‐N in HL‐1 (G) and quantitative data (H). (I) Schematic illustrating that USP25 activates the NLRP3 inflammasome via deubiquitinating NLRP3 at residue K243 by its active site C178. Significance is defined as **p < .01, ****p < .0001. The abbreviation ‘NS’ denotes no statistical significance (*p* > .05).

To identify the deubiquitination site of NLRP3 mediated by USP25, we performed a Co‐IP‐based ubiquitinated peptide enrichment analysis in HL‐1 cells expressing Flag‐USP25 (Figure 7A). As shown in Figure 7B, we identified seven potential ubiquitination lysine residues—K22, K24, K34, K243, K543, K557 and K597—in NLRP3 protein and constructed these seven NLRP3 mutants (mutation of lysine to arginine). Among these seven mutants, only NLRP3^K243R^ showed increased cell viability against H/R‐induced pyroptosis in HL‐1 cells, compared to NLRP3^WT^, suggesting that the protein modification at K243 site may reverse the H/R‐induced cardiomyocyte injury (Figure 7C). Moreover, the polyubiquitination of NLRP3^K243R^ was much lower than NLRP3^WT^ and was not further reduced by USP25 (Figure 7D), suggesting that K243 is required for USP25‐mediated NLRP3 deubiquitination. As expected, NLRP3^K243R^ significantly inhibited H/R‐induced release of LDH and IL‐1β in cardiomyocytes, and USP25 could not reduce LDH/IL‐1β release when K243 in NLRP3 was mutated (Figure 7E,F). In addition, H/R stimulation significantly enhanced the protein expression levels of Cleaved Caspase‐1 and GSDMD‐N in myocytes overexpressing NLRP3 (Figure S7). However, the K243R mutation of NLRP3 inhibited the activation of NLRP3 inflammasomes, and USP25 failed to further inactivate NLRP3 in HL‐1 cells with NLRP3^K243R^ (Figure 7G,H). Summarily, USP25 deubiquitinates NLRP3 at K243 via its active site C178 to block NLRP3 inflammasome activation in cardiomyocytes (Figure 7J).

### 3.8 USP25 failed to ameliorate I/R‐induced cardiac injury in NLRP3 knockout mice

**FIGURE 8:**
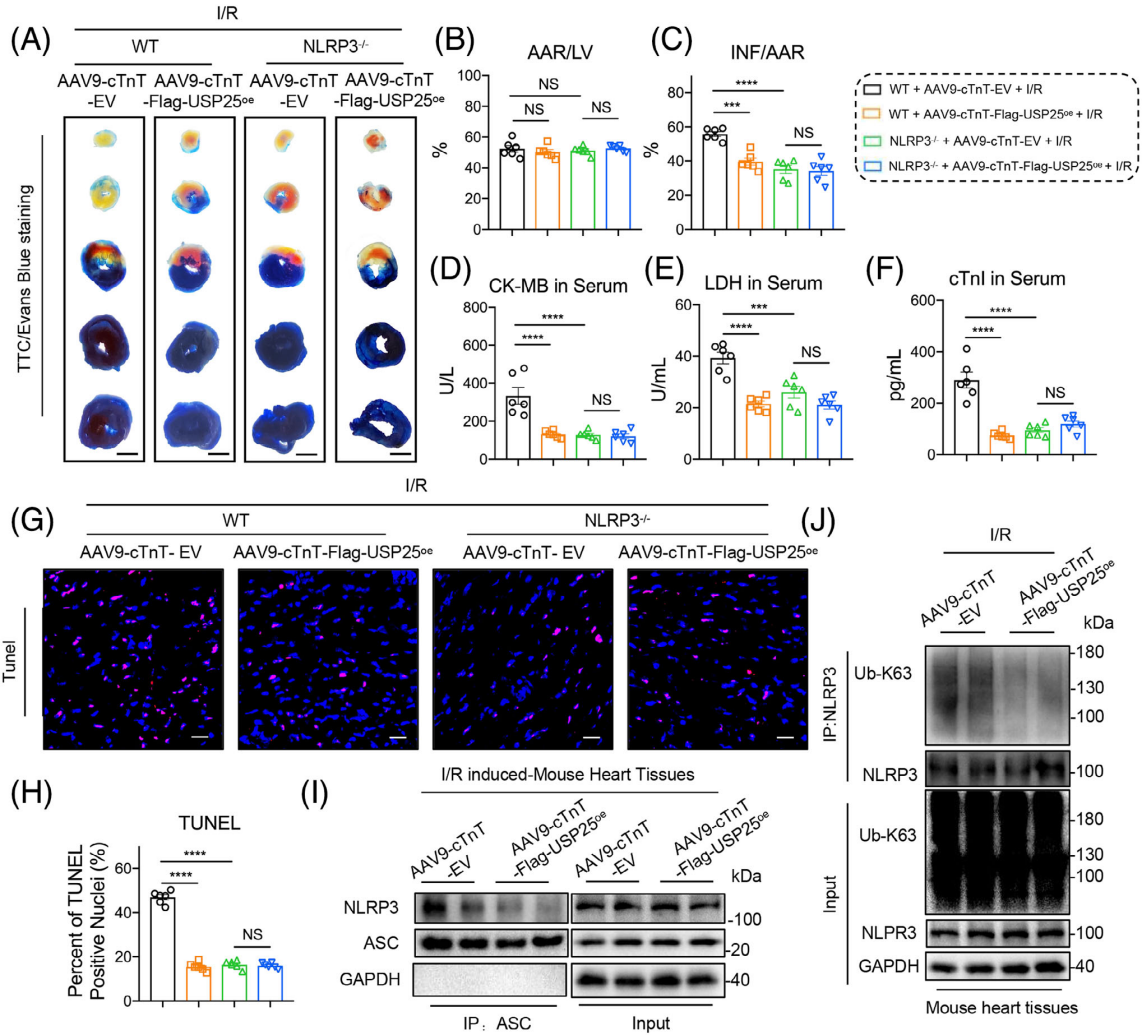
Ubiquitin‐specific protease 25 (USP25) failed to ameliorate ischemia/reperfusion (I/R)‐induced cardiac injury in NLRP3 knockout mice. The whole‐body NLRP3 knockout (NLRP3^−/−^) mice and wild‐type littermates were injected with AAV9‐cTnT‐Flag‐USP25^oe^ via tail vein (1 × 10E + 11 v.g./mouse) 4 weeks before I/R surgery (30 min ischaemia followed by 4 h reperfusion) and sham operation. (A–C) Representative images of sections stained with Evans Blue (EB) and 2,3,5‐triphenyltetrazolium chloride (TTC) (A) and quantitative data for area at risk (AAR) and left ventricular (LV) area (B) and infarcted area size (INF) and AAR (C). (D and E) The content of creatine kinase (CK)‐MB (D), lactate dehydrogenase (LDH) (E) and cTnI (F) in serum from each group. (G and H) Representative images of sections stained with TUNEL in the infarcted area (red: positive cells; scale bar, 1 mm and 100 µm; G) and quantitative data (H). (I) Co‐immunoprecipitation of endogenous ASC and NLRP3 in I/R‐induced heart tissues from wild‐type mice that were injected with AAV9‐cTnT‐EV or AAV9‐cTnT‐Flag‐USP25^oe^. Endogenous ASC was immunoprecipitated by anti‐ASC antibody. Significance is defined as ***p < .001, ****p < .0001. The abbreviation ‘NS’ denotes no statistical significance (*p* > .05). (J) Immunoprecipitation of NLRP3 in I/R‐induced heart tissues from wild‐type mice that were injected with AAV9‐cTnT‐EV or AAV9‐cTnT‐Flag‐USP25^oe^. Ubiquitinated NLRP3 was detected by immunoblotting using an Ub‐K63 antibody to clarify the K63 ubiquitination level of NLRP3 regulated by USP25.

To verify that USP25 improves myocardial I/R injury by inactivating NLRP3, the whole‐body NLRP3 knockout (NLRP3^−/−^) mice were used. The recombinant AAV9 vector carrying USP25 and including a cardiac‐specific promoter cTnT was manufactured and administered to WT or NLRP3^−/−^ mice via tail vein injection to overexpress USP25 specifically in cardiomyocytes (Figure S8). We verified and confirmed the successful overexpression of Flag‐tagged USP25 in the myocardial tissues of mice with AAV9 administration (Figure S9). TTC/Evan Blue staining assay showed the reduced myocardial infarction area in I/R‐treated USP25^oe^ mice, indicating that cardiomyocyte‐specific USP25 overexpression significantly ameliorated myocardial I/R injury (Figure 8A–C). However, USP25 overexpression lost its ability to protect hearts against MI/RI in NLRP3^−/−^ mice (Figure 8A–C). Examinations on cardiac injury markers, including serum CK‐MB, LDH and cTnI levels, showed similar results (Figure 8D–F). As expected, cardiomyocyte‐specific USP25 overexpression decreased the TUNEL‐positive cells in myocardium tissues of I/R mice but failed when the NLRP3 was knocked out (Figure 8G,H). In addition, we observed that cardiomyocyte‐specific USP25 overexpression significantly reduced the protein levels of Cleaved Caspase‐1 and GSDMD‐N in I/R‐induced mouse heart tissues (Figure S10). In addition, this overexpression resulted in decreased K63‐linked polyubiquitination of NLRP3 and NLRP3–ASC interaction in I/R‐induced mouse heart tissues (Figure 8I–J). Furthermore, we conducted in vitro experiments and confirmed that the NLRP3 inhibitor MCC950 abolished the enhancement of cardiomyocyte activity induced by USP25 under H/R stimulation (Figure S11A), as well as its anti‐pyroptosis effects (Figure S11B,C). These findings reinforce the significance of the USP25–NLRP3 axis in mitigating MI/RI.

## 4 DISCUSSION

The study uncovers the protective role of cardiomyocyte USP25 against MI/RI. We found that the expression level of USP25 was significantly decreased in I/R‐induced mouse heart tissues and primary cardiomyocytes in a time‐dependent manner. USP25 deficiency exacerbates MI/RI and aggravates I/R‐induced cardiac remodelling in mice. Mechanistically, USP25 directly binds to NLRP3 protein and K63‐linkedly deubiquitinates NLRP3 at residue K243 via its active site C178, thus affecting NLRP3–ASC interaction and ASC oligomerization to inhibit NLRP3 activation and pyroptosis in cardiomyocytes. We further showed that the overexpression of USP25 in cardiomyocytes ameliorated MI/RI in mice, whereas this protective effect disappeared when NLRP3 was knocked out. In this study, we identified a DUB, USP25, that targets NLRP3 for the treatment of MI/RI and provided a new perspective for our understanding of the self‐protection mechanism of the heart in MI/RI. Collectively, our study successfully identifies NLRP3 as a substrate protein of USP25 and highlights the beneficial role of USP25 in protecting hearts from MI/RI.

Previous studies have shown that USP family members are associated with I/R injury of liver,^30^ kidney,^31^ brain,^32^ spinal cord^33^ and intestine.^34^ In the present study, we showed that USP25 expression was down‐regulated in I/R‐induced mouse heart tissues and confirmed that cardiomyocyte‐derived USP25 was involved in MI/RI as a protective factor. Interestingly, our group has recently reported that USP25 improves pathological myocardial hypertrophy by deubiquitinating SERCA2a and increasing SERCA2a stability in cardiomyocytes.^19^ These studies show together that cardiomyocyte USP25 plays a protective role in different heart diseases via deubiquitinating respective substrate proteins, indicating that USP25 may have a broad‐spectrum application for treating heart diseases. Therefore, the exogenous supplementation of UPS25 in cardiomyocytes should be a potential therapeutic strategy for the treatment of heart diseases.

Activation of NLRP3 inflammasome promotes Caspase‐1 activation, which converts pro‐IL‐1β and pro‐IL‐18 into their active forms.^35^ In addition, the polymerization of GSDMD‐N from GSDMD that was cleaved by Caspase‐1 formed pores in the plasma membrane, resulting in loss of membrane integrity, release of IL‐1β and IL‐18 and ultimately leading to pyroptosis.^36^ It has been reported that NLRP3 activation and NLRP3‐mediated pyroptosis are closely related to the I/R‐induced cardiomyocyte injury and the aggravation of MI/RI,^37^ and inhibition of NLRP3 significantly alleviates MI/RI via down‐regulating cardiomyocyte pyroptosis.^38^ However, designing and discovering small‐molecule inhibitors of NLRP3 with high selectivity, efficiency and druggability has proved to be painstaking.^39^ The shrimp‐shaped NLRP3 protein lacks an obvious binding pocket that allows the compound to lock in, and worst of all, no complete structure of NLRP3 inflammasome has been reported. MCC950, a NLRP3 selective inhibitor first reported by O'Neil and Cooper, is considered the best one to date.^40^ However, the exact binding mechanism of this compound with NLRP3 remains a mystery, which has limited the clinical trials of MCC950 and the development of better drug candidates based on MCC950.^41^ Currently, NLRP3 inhibitor dapansutrile^42^ is being evaluated in Phase II clinical trial for treating gout flares. However, NLRP3 inhibitors have yet to be utilized in cardiovascular diseases. Given that NLRP3 is extensively expressed in various organs and tissues, systemic administration of inhibitors targeting NLRP3 may result in significant immunosuppression, posing potential side effects. Furthermore, traditional small‐molecule inhibitors may exhibit multiple targets. Thus, regulating NLRP3 via DUBs may provide new therapeutic strategies for NLRP3‐driven diseases. Here, we demonstrate that USP25 deubiquitinates NLRP3 to inhibit NLRP3 activation in cardiomyocytes. Therefore, supplementing USP25 in hearts via gene induction may supply a new way to limit NLRP3 activation and then attenuate NLRP3‐driven MI/RI. Another way utilizing USP25 to regulate NLRP3 is the USP25–NLRP3‐based deubiquitinase‐targeting chimaera (DUBTAC), which may offer a precise approach to link USP25 and NLRP3 together and then inhibit NLRP3 activity in the cells. Because the DUBTAC specifically recognizes the target DUB and the substrate, it may minimize the off‐target side effects. Thus, it may be a strategy for medicinal chemists to design small‐molecule DUBTACs targeting both USP25 and NLRP3 with high druggability for the treatment of MI/RI. However, one of the difficulties of this technique is that the designed DUBTAC that binds to the DUB should retain the catalytic function of the DUB. Here, we find that the UBA domain of USP25 binds to NLRP3, and the USP domain of USP25 deubiquitinates NLRP3, indicating that the binding domain and catalytic domain of USP25 towards NLRP3 are independent. This mode makes designing DUBTAC for USP25–NLRP3 axis more convenient.

Ubiquitination of NLRP3 plays a key role in regulating the stability and activity of NLPR3. It has been reported that a variety of DUBs can modify NLRP3, including UAF1,^43^ USP22,^44^ OTUD6a,^45^ USP19^46^ and USP14.^47^ Interestingly, all these reported DUBs regulate the protein stability of NLRP3 through the K48‐linked ubiquitination–proteasome degradation pathway. A E3 ubiquitin ligase, Cullin1, binds to NLRP3 and promotes K63‐linked ubiquitination at K689 site, which inhibits the interaction between NLRP3 and ASC and thus impedes the assembly of NLRP3 inflammasome.^48^ However, another E3 ubiquitin ligase, Pellino2, activates NLRP3 by promoting K63‐linked ubiquitination of NLRP3 protein.^49^ Therefore, the regulatory mechanism of the K63‐linked ubiquitination modification of NLRP3 activity is complex. So far, no DUBs have been reported to regulate NLRP3 activity. In this study, we determined that USP25 showed no effect on the NLRP3 protein level in cardiomyocytes in the absence of I/R and H/R. It was found that USP25 is able to remove ubiquitin molecules linked to the NLRP3 protein through K63‐linked ubiquitination, indicating USP25 regulates NLRP3 activity. NLRP3–ASC interaction and ASC oligomerization are two key steps and hallmarks of NLRP3 activation, which then advances to subsequent pyroptosis. Therefore, we examined if USP25 influenced NLRP3–ASC interaction and ASC oligomerization. Notably, our experiments confirmed that USP25 indeed affects the interaction between NLRP3 and ASC and the subsequent ASC oligomerization. These data suggest that K63‐linked ubiquitination modification of NLRP3 by USP25 prevents NLRP3–ASC interaction and subsequent NLRP3 activation. This is the first time to report that DUB regulates NLRP3 activity, rather than protein stability. Subsequently, we demonstrate for the first time that USP25 inhibits the activation of NLRP3 by removing the ubiquitin from the K243 residue of NLRP3, suggesting K243 as a new functional residue for regulating NLRP3 activity. Our findings clearly illustrate how a DUB, USP25, negatively regulates NLRP3 activity through deubiquitinating it. Given that USP25 is associated with multiple substrate proteins, it may also play a role in the regulation of MIRI through these substrates. This possibility warrants further investigation to enhance our understanding of the molecular network involved in USP25's regulation of MI/RI.

A limitation of this study is that we did not employ the cardiomyocyte‐specific USP25 knockout mice. However, our in vitro studies using primary cardiomyocytes and the in vivo experiments using mice with cardiomyocyte‐specific USP25 overexpression supported the conclusion that cardiomyocyte USP25–NLRP3 axis mediates MI/RI. We also acknowledge that the roles of USP25 in fibroblasts, endothelial cells and infiltrated immune cells during MI/RI are also interesting and deserve further investigation. In addition, we do not know how I/R stimuli down‐regulate USP25 expression in cardiomyocytes. This should involve the negative regulation of the upstream transcriptional factor of USP25 gene. Future research should aim to identify the upstream transcription factor of USP25, elucidate its regulatory mechanisms and develop pharmacological agents based on this transcription factor. Such efforts could enhance the in vivo expression of USP25 protein, thereby contributing to the treatment of MI/RI. Furthermore, it must be noted that USP25 is associated with multiple substrate proteins; we could not completely exclude the possibility of other substrates participating in the cardioprotective effects of USP25 against MI/RI. In addition, it should be noted that USP25‐mediated K243 deubiquitination of NLRP3 occurs in the NACHT domain, whereas ASC interacts with the LRR domain of NLRP3 protein. The mechanism by which USP25‐mediated deubiquitination of the NLRP3 NACHT domain impedes the interaction between the ASC and NLRP3 LRR domains warrants further investigation. Moreover, the evidence that K243 of NLRP3 is involved in the regulation of NLRP3‐mediated pyroptosis would be even stronger if the NLRP3 wild type and various mutant plasmids were introduced into endogenous NLRP3 knockout cells. Finally, in clinical practice, accurately predicting the timing of MI/RI remains challenging. Our experimental results indicate that overexpression of USP25 in mice prior to I/R has an observable effect on MI/RI. However, it is essential to further evaluate whether the therapeutic effects of USP25 overexpression persist in vivo after reperfusion. Additionally, we must refine the toxicological studies of AAV9‐cTNT‐USP25 to provide reliable safety data for future clinical applications of this gene therapy.

In conclusion, we demonstrate that USP25 ameliorates MI/RI by deubiquitinating NLRP3 and limiting NLRP3 activity and the subsequent cardiomyocyte pyroptosis. This work extends our understanding of the role of USP25 in MI/RI and presents USP25 as a new DUB regulating NLRP3 activity through K63‐linked deubiquitinating modification at K243 site. Increasing or inducing USP25 levels in heart may be a promising approach for the treatment of MI/RI.

## AUTHOR CONTRIBUTIONS

Guang Liang, Wenhai Huang and Bozhi Ye contributed to the literature search and study design. Bozhi Ye, Diyun Xu, Lingfeng Zhong, Haowen Xu, Xue Han and Julian Min performed the experiments and analysed the data. Wei Wang and Gaojun Wu provided technical help. Bozhi Ye, Yi Wang and Guang Liang participated in the drafting of the article. All authors agree to be accountable for all aspects of work, ensuring integrity and accuracy.

## CONFLICT OF INTEREST STATEMENT

The authors declare no conflicts of interest.

## ETHICS STATEMENT

All the experiments involving human samples were approved by the Ethics Committee of The First Affiliated Hospital of Wenzhou Medical University (Wenzhou, China; approval no. KY2022‐156) and conformed to the principles outlined in the Declaration of Helsinki. All animal husbandry protocols, experimental procedures and animal welfare measures were conducted in accordance with the guidelines and regulations approved by the Ethics Committee of the Laboratory Animal Center of Wenzhou Medical University (No. WYYY‐AEC‐YS‐2022‐0337).

## Supporting information



Supporting Information

## Data Availability

All data needed to evaluate the conclusions in this study are presented in this manuscript or the Supporting Information section. The materials described in this study are either commercially available or available upon reasonable request from the corresponding authors.
