# Supplementary material for: Ubiquitin‐specific protease 25 improves myocardial ischemia–reperfusion injury by deubiquitinating NLRP3 and negatively regulating NLRP3 inflammasome activity in cardiomyocytes
Source: Clin Transl Med. 2025 Feb 22;15(2):e70243. doi: 10.1002/ctm2.70243 (PMC11845855; doi:10.1002/ctm2.70243)
Supplement: Supplementary file 1 — Supporting Information [file CTM2-15-e70243-s001.docx]

***Supplementary Information***

**Ubiquitin-specific protease 25 improves myocardial ischemia-reperfusion injury by deubiquitinating NLRP3 and negatively regulating NLRP3 inflammasome activity in cardiomyocytes**

**Contents in Supplementary File**

Supplementary 2 Tables, 11 Figures, and Legends

***Supplementary Tables:***

**Supplementary Table S1:** The detailed information regarding plasmids used in the study.

| Clone Name | Source /  Repository | Persistent ID / URL |
| --- | --- | --- |
| Plasmids-Rat-USP25 | NM_001107114 | Purchased from Shanghai Genechem Co.,Ltd. |
| Plasmids-Mouse- USP25(K462samesense) | NM_013918 | Purchased from Shanghai Genechem Co.,Ltd. |
| Plasmids-Mouse- USP25(del14-57aa) | NM_013918 | Purchased from Shanghai Genechem Co.,Ltd. |
| Plasmids-Mouse- USP25(del14-57/97-116aa) | NM_013918 | Purchased from Shanghai Genechem Co.,Ltd. |
| Plasmids Mouse- USP25(del14-57/97-116/123-140aa) | NM_013918 | Purchased from Shanghai Genechem Co.,Ltd. |
| Plasmids- Mouse- USP25(del169-658aa) | NM_013918 | Purchased from Shanghai Genechem Co.,Ltd. |
| Plasmids-Mouse- USP25(C178A) | NM_013918 | Purchased from Shanghai Genechem Co.,Ltd. |
| Plasmids-Mouse- USP25(H608A) | NM_013918 | Purchased from Shanghai Genechem Co.,Ltd. |
| Plasmids-Mouse- NLRP3-WT | NM_145827.4 | Purchased from Shanghai Genechem Co.,Ltd. |
| Plasmids-Mouse- NLRP3-PYD(1-91aa) | NM_145827.4 | Purchased from Shanghai Genechem Co.,Ltd. |
| Plasmids-Mouse- NLRP3-NACHT(216-532aa) | NM_145827.4 | Purchased from Shanghai Genechem Co.,Ltd. |
| Plasmids-Mouse- NLRP3-LRR(739-988aa) | NM_145827.4 | Purchased from Shanghai Genechem Co.,Ltd. |
| Plasmids- Mouse-ASC | NM_023258.4 | Purchased from Shanghai Genechem Co.,Ltd. |
| Plasmids- Mouse-Ub/K63-Ub | [NM_001313984.2](https://www.ncbi.nlm.nih.gov/nuccore/NM_001313984.2) | Purchased from Shanghai Genechem Co.,Ltd. |
| Plasmids- Mouse- NLRP3-K22R | NM_145827.4 | Purchased from Nanjing GenScript Co.,Ltd. |
| Plasmids- Mouse-NLRP3-K24R | NM_145827.4 | Purchased from Nanjing GenScript Co.,Ltd. |
| Plasmids- Mouse-NLRP3-K34R | NM_145827.4 | Purchased from Nanjing GenScript Co.,Ltd. |
| Plasmids- Mouse-NLRP3-K243R | NM_145827.4 | Purchased from Nanjing GenScript Co.,Ltd. |
| Plasmids- Mouse-NLRP3-K543R | NM_145827.4 | Purchased from Nanjing GenScript Co.,Ltd. |
| Plasmids- Mouse-NLRP3-K557R | NM_145827.4 | Purchased from Nanjing GenScript Co.,Ltd. |
| Plasmids- Mouse-NLRP3-K597R | NM_145827.4 | Purchased from Nanjing GenScript Co.,Ltd. |

**Supplementary Table S2:** Sequences of primers for qPCR used in the study.

| **Gene** | **Species** | **FW** | **RW** |
| --- | --- | --- | --- |
| *Usp1* | Mouse | TGCTTTGCTGCTAGTGGCTTGG | TCGTCGGCTTTGTGCTCCATTC |
| *Usp2* | Mouse | CTCGGCTGGCTTCTCTCGTTTG | GGTGGGCTGACGGTCCTCTC |
| *Usp3* | Mouse | ACAGTGTGTATGGATTGCAGTAG | CCTGTCCGCTGTAAAGGCT |
| *Usp11* | Mouse | GGCTGTATCAACAATGCTGGG | TCATCTCCTTCTAGCAGTCTCTC |
| *Usp12* | Mouse | CAGTCTCCAAATTCGCCTCCA | GTGCTCGTTGACCGGAAACT |
| *Usp13* | Mouse | GACCGCGTCTACAAGAACGAG | CTTTCTACGTGTTCCCTTCCAAA |
| *Usp15* | Mouse | GACGCTGCTCAAAACCTCG | CGATGGGTCCAGGATAGACATT |
| *Usp16* | Mouse | AGAAACGGACCAAGGGGAGAA | AGAGCCAAACCGAAGGGTCT |
| *Usp18* | Mouse | GACCAGATCACGGACACAGACTTG | GTTTGCTCCTCCTGCTGCTCTC |
| *Usp19* | Mouse | TGAACCAGAGCAGTGTACGTT | CTGCACCTCGTGTAGCAGG |
| *Usp21* | Mouse | AACTCCATGTTACGACCTTTGC | AAGGGGACCTCTAGGACGAGA |
| *Usp24* | Mouse | ACATTAGCATACGCCACAGCCTTG | GGAATCCCATCGAGGTGATAAT |
| *Usp25* | Mouse | GTGTTACCGACGATCCGTGTC | GTTCATGCAGATATAGAGGCCAC |
| *Usp27x* | Mouse | GACATTGAGCAAATTGCCAAAGA | AAGTCCCGGCACTGAACAC |
| *Usp28* | Mouse | CTCTTCTTCGTCAGGTGCCTTATCG | GGTTGTAGATGTATGCCCAGTAGTGTC |
| *Usp29* | Mouse | TCCGCAGCACAAACAGGAG | CTCACCACTAACCACTACGCC |
| *Usp30* | Mouse | GGGAGTGATCGGTGGGATTG | TCTTCCTCTCTGTAATGGGACC |
| *Usp31* | Mouse | GGAGTACACCCCGCAACAC | TGCTTTACAGCATGGTTGAGG |
| *Usp32* | Mouse | GGAAATGCCTGTATTGCTGTGGATTG | CTTGTGCTCGCCGACTCTGTTC |
| *Usp35* | Mouse | AAGGAGCTGTTCGCTGTCATC | TCATCCTGCTAATGGCAGTCA |
| *Usp37* | Mouse | TGCAGACTGGGATTACAAAGTG | ACGAGCAGGCTGACTCTATTG |
| *Usp40* | Mouse | CACTATGCCATGTGCCTCCTCTTG | GCTTCTTTGATGGTCCCGTCTCTG |
| *Usp43* | Mouse | GCAGAGGAGGTGATCTTGGTTGAAC | CTTGTGCTCGCCGACTCTGTTC |
| *Usp46* | Mouse | ATGACTGTCCGAAACATCGCC | TTGACCAATCCGAAGTAGTGTTC |
| *Usp49* | Mouse | GGACGAACGCAGAACATACACATTG | ACAGAGCAGGAATCATAGGGCATTG |
| *Usp50* | Mouse | GTTACCACCGCCTTTGCCTACC | TGCCAACAGCCGACAGAAATATCTC |
| *Usp53* | Mouse | AAGCCTAGCGGCAATCTTGG | GTTCTGCCCTGGCTCGTTTA |
| *Usp54* | Mouse | TTCTGGAAGCCGAGGTAGTGT | GGCGGAAGATGTCCAAGTG |
| *Uchl3* | Mouse | TACCCTTCCTTCTATCCAACCTC | TCAGTAAAACCGAAGCCCTTTC |
| *Uchl5* | Mouse | CTCATTAAAGGATTCGGTTGCCG | AGTACACTTACGATAGCCTGAGT |

**
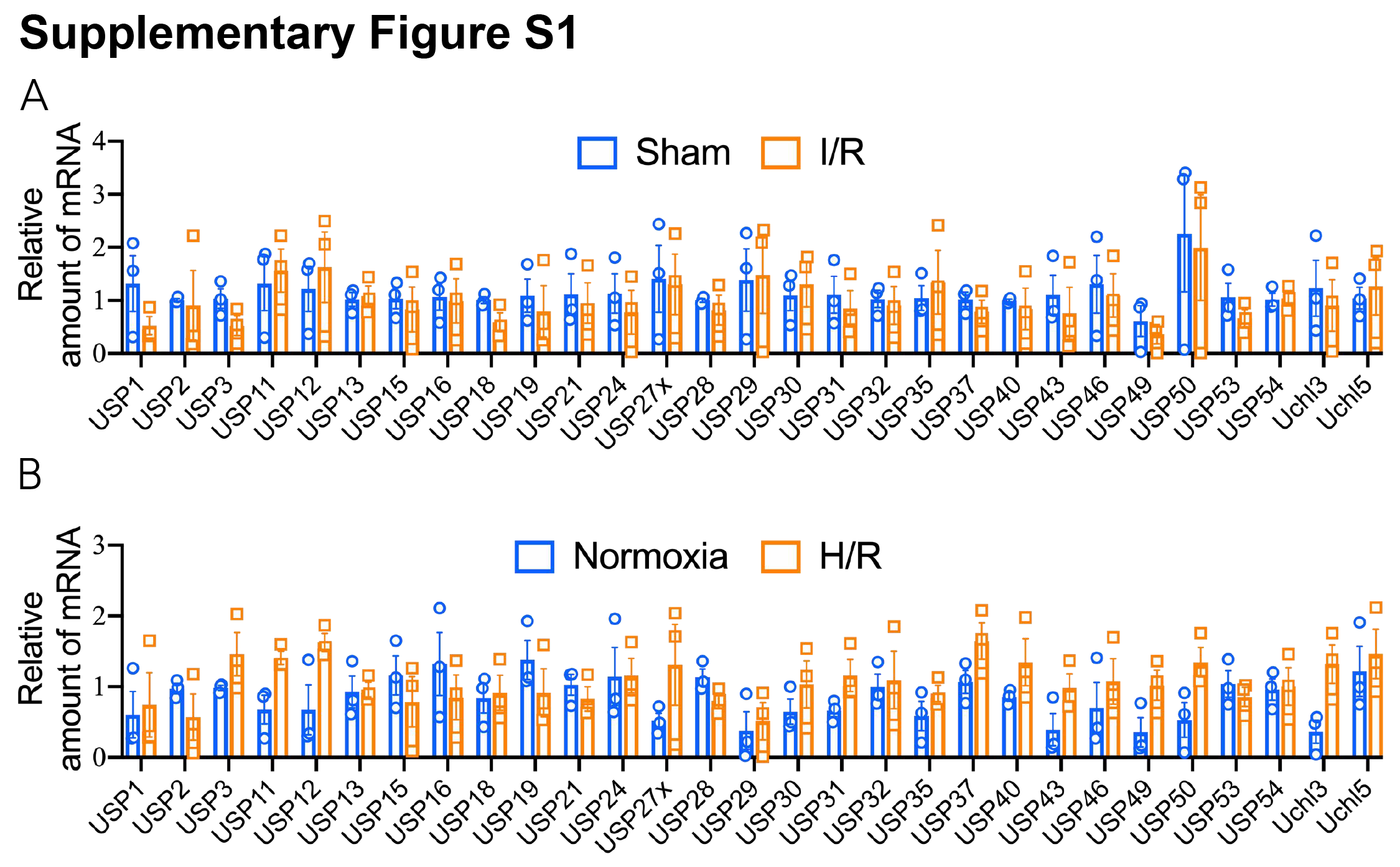
Supplementary Figure S1:**

(**A-B**) RT-qPCR analysis of the mRNA expression of DUBs of USP family members that were differentially expressed in RNA transcriptome sequencing induced by myocardial I/R injury (A) and cardiomyocyte hypoxia/reoxygenation (H/R) injury (B). n=3.

**
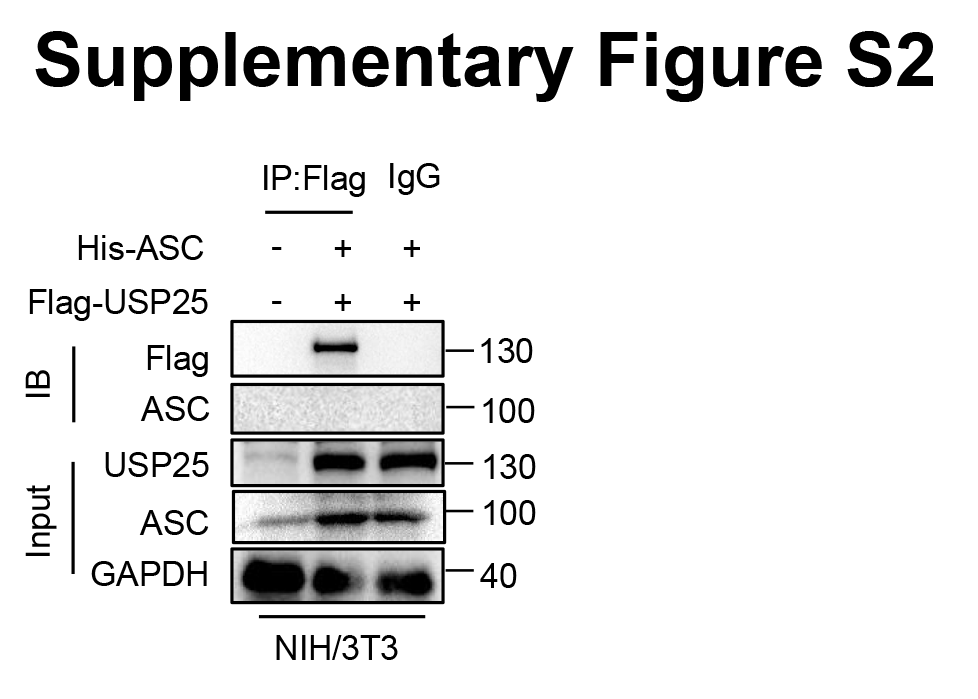
**

**Supplementary Figure S2:**

Coimmunoprecipitation of USP25 and ASC in NIH/3T3 cells co-transfected with Flag-USP25 and His-ASC plasmids. Exogenous USP25 was immunoprecipitated by anti-Flag antibody.

**
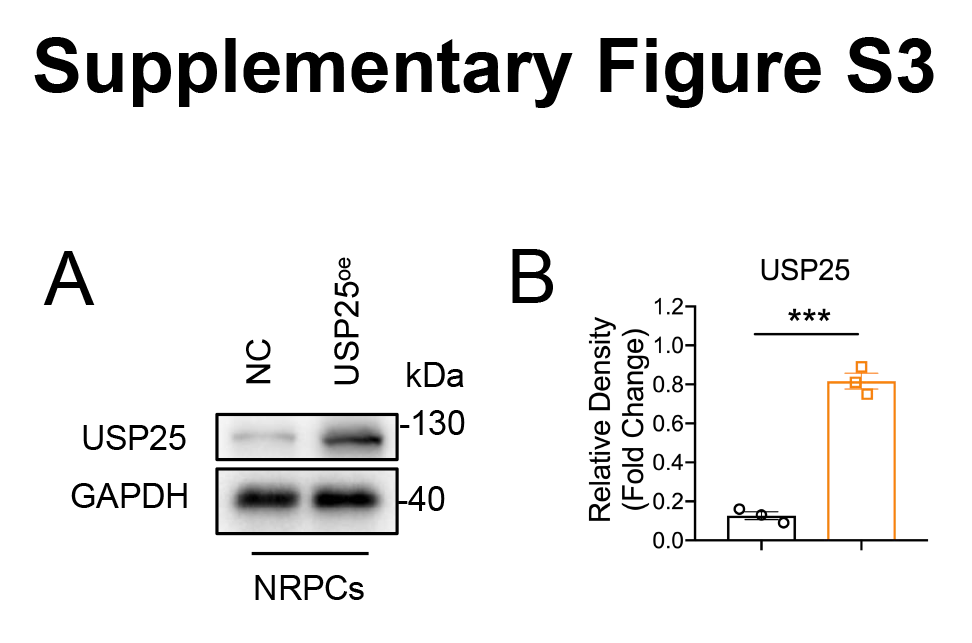
**

**Supplementary Figure S3:**

**(A-B)** USP25 overexpression in NRPCs was carried out by transfection of plasmids, the expression level of USP25 protein was measured by Western blot (A) and statistical results (B) (n = 3). Significance is defined as ****P* < 0.001.

**
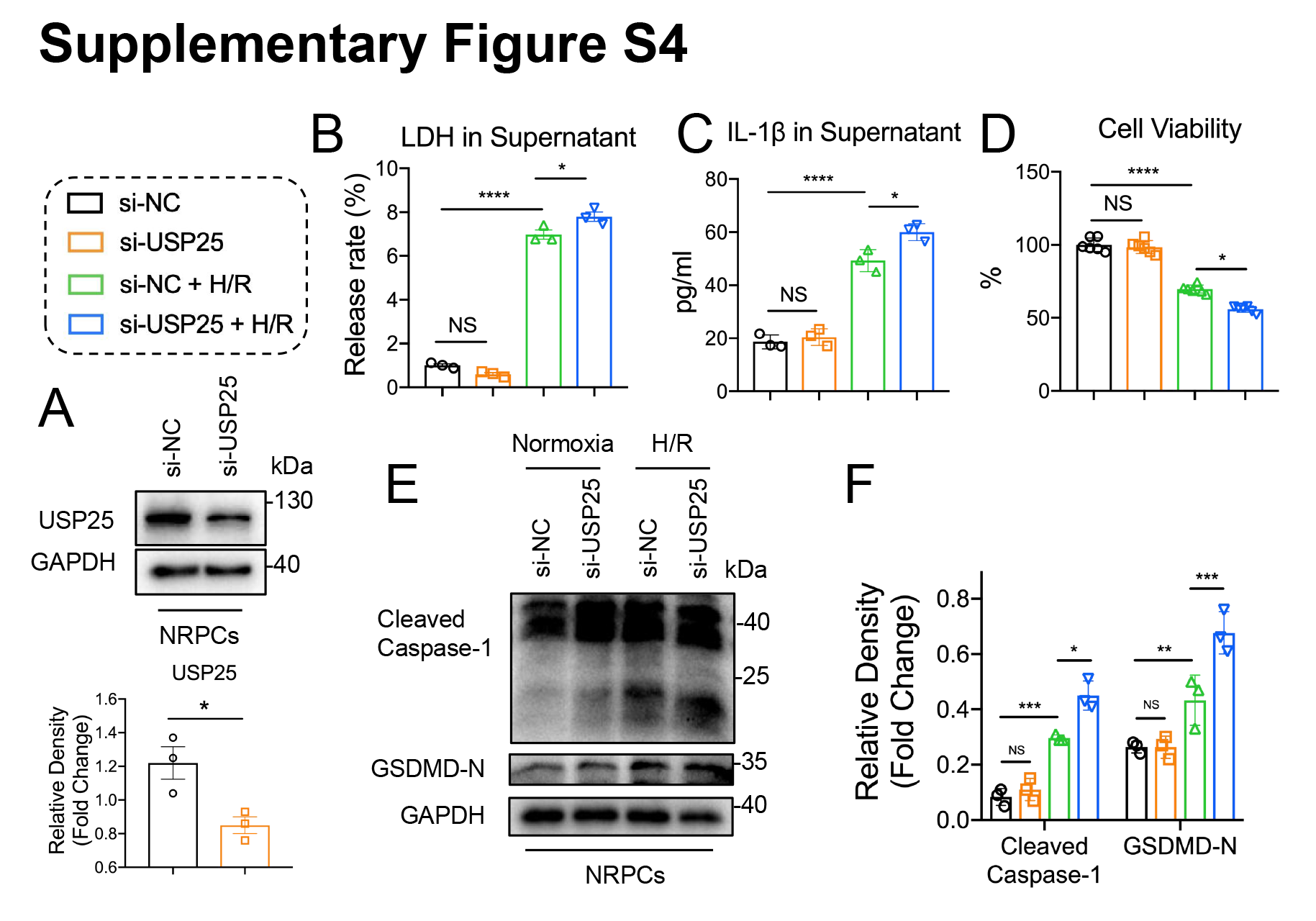
**

**Supplementary Figure S4:**

NRPCs were subject to H/R injury (4 h hypoxia followed by 6 h reoxygenation) after transfection with siRNA.

**(A)** Representative western blot results of USP25 in NRPCs with siRNA transfection.

**(B-C)** The release of LDH (B) and IL-1β (C) in culture medium.

**(D)** Cell viability of NRPCs.

**(E-F)** Representative western blot results of Cleaved Caspase-1 and GSDMD-N in NRPCs (E) and quantitative data (F).

Significance is defined as **P* < 0.05, ***P* < 0.01, ****P* < 0.001, *****P* < 0.0001. The abbreviation "NS" denotes no statistical significance (*P* ＞ 0.05).

**
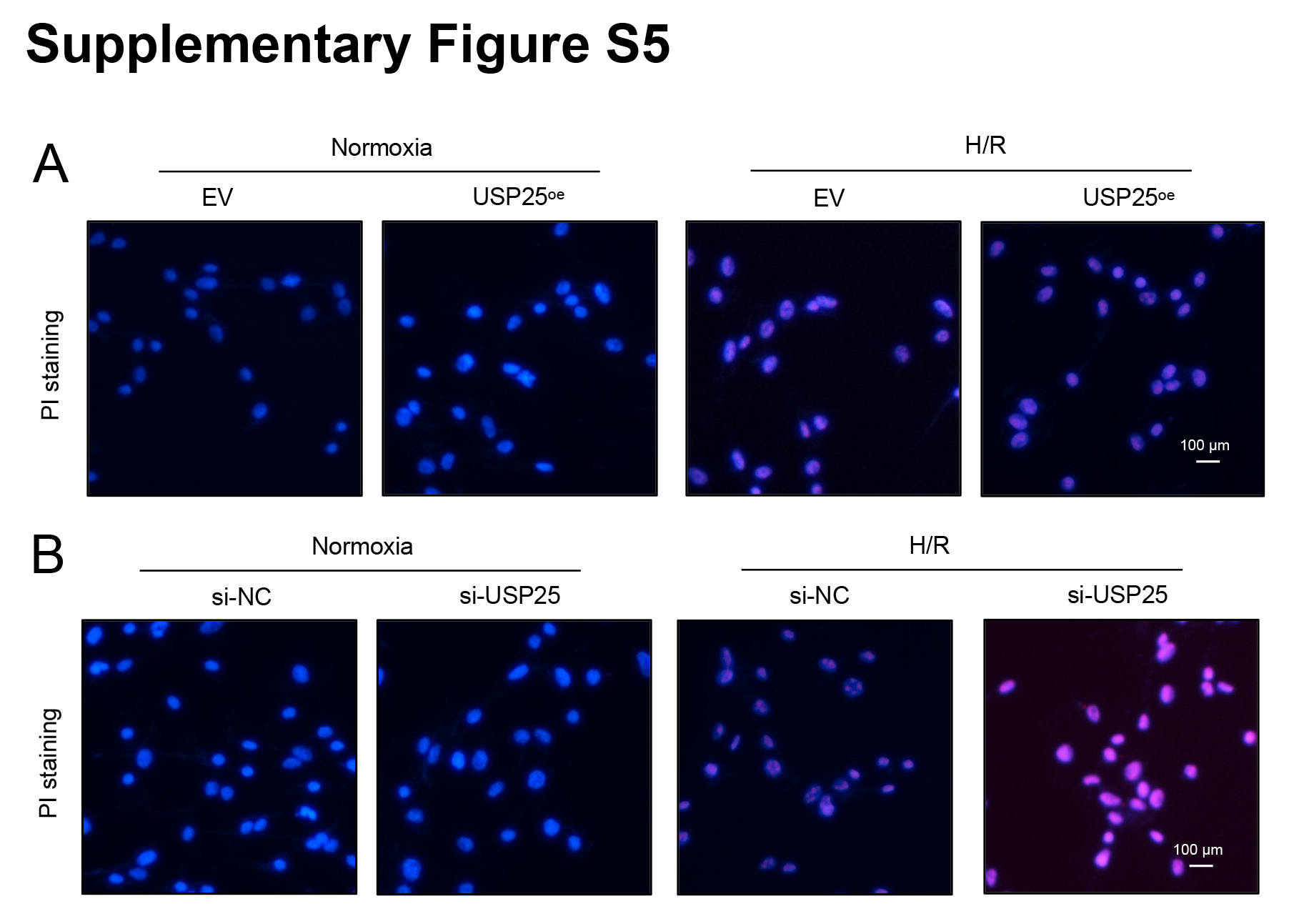
**

**Supplementary Figure S5**

NRPCs were subject to H/R injury (6 h LPS followed by 30 min Nigricin) after transfection with siRNA or plasmids.

**(A)** Representative images of PI staining in NRPCs with plasmid transfection (scale bar, 100 μm).

**(B)** Representative images of PI staining in NRPCs with siRNA transfection (scale bar, 100 μm).

**
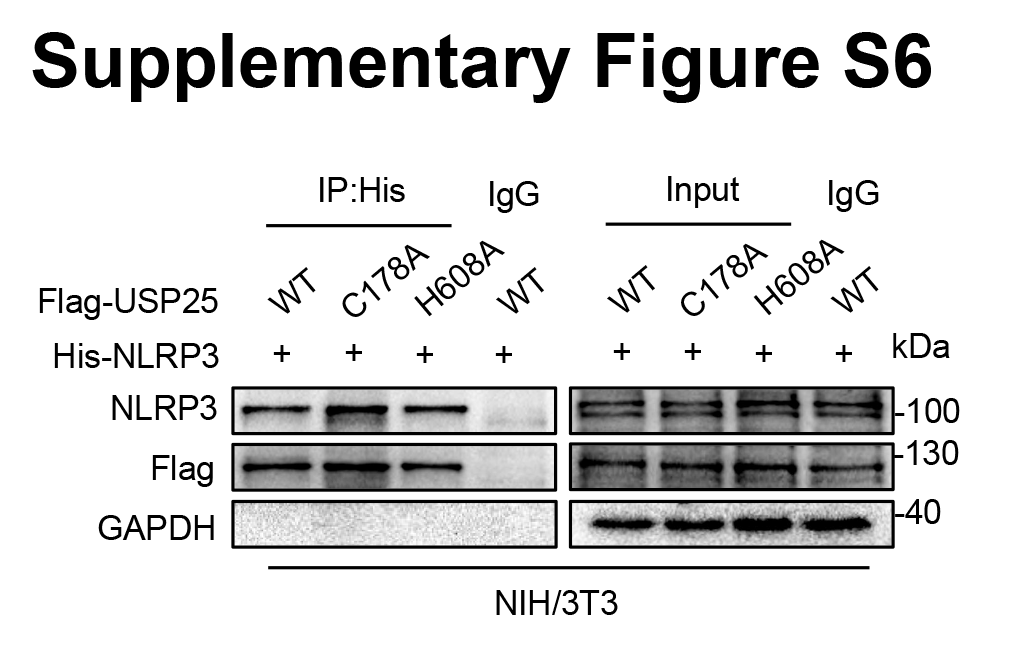
**

**Supplementary Figure S6:**

Coimmunoprecipitation of USP25^C178A^, Flag-USP25^H608A^ and NLRP3 in NIH/3T3 cells co-transfected with Flag-USP25^C178A^, Flag-USP25^H608A^ and His-NLRP3 plasmids. Exogenous normal or mutated USP25 was immunoprecipitated by anti-Flag antibody. IgG, immunoglobulin G.

**
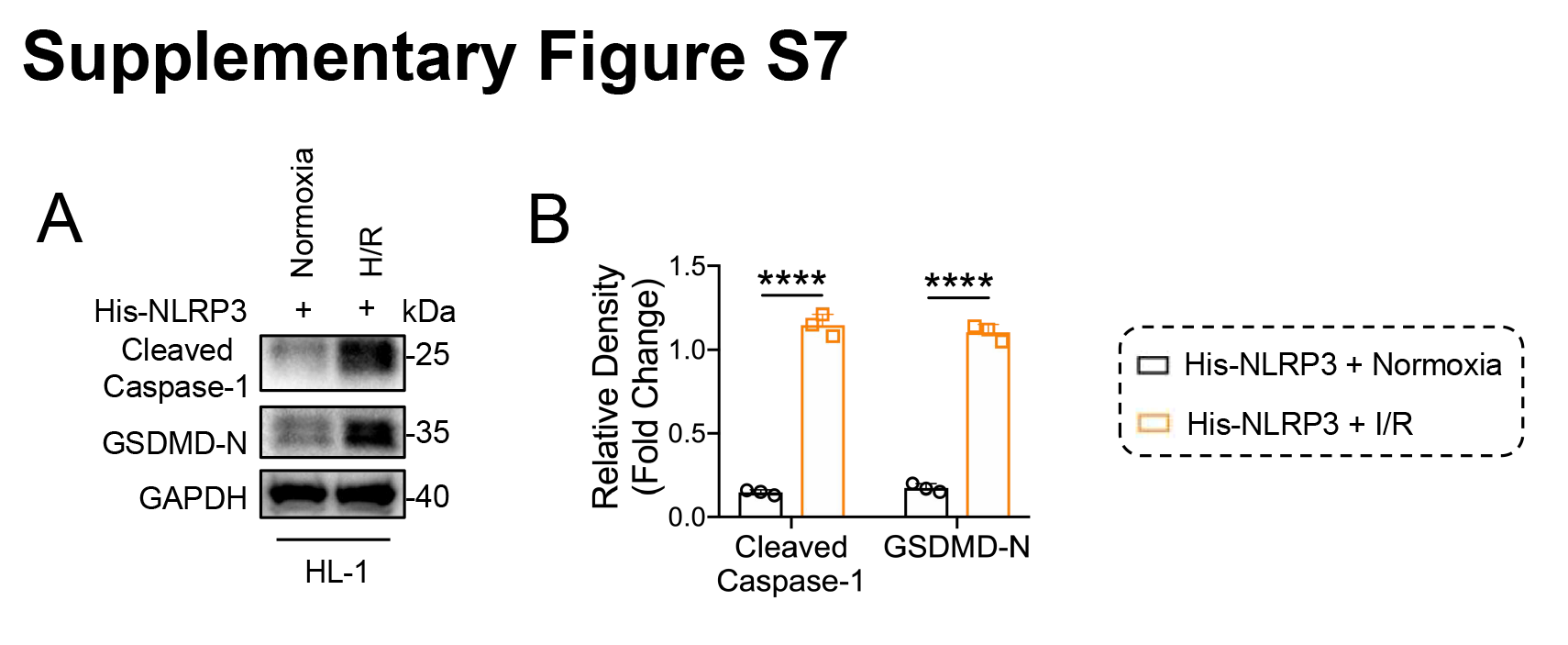
**

**Supplementary Figure S7:**

(**A-B**) Representative western blot results of Cleaved Caspase-1 and GSDMD-N in H/R-induced HL-1 transfected with His-NLRP3 (A) and quantitative data (B). P < 0.0001. n = 3.

**
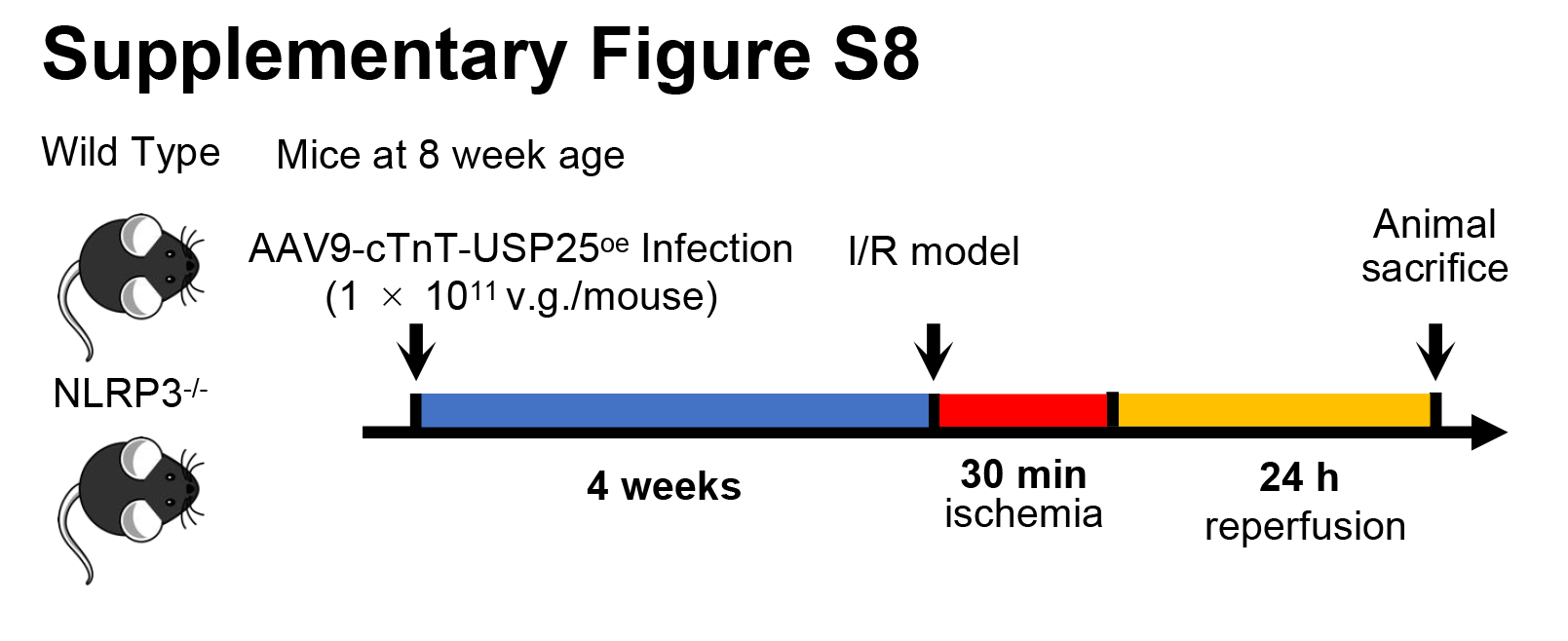
**

**Supplementary Figure S8:**

Schematic representation of the establishment of the I/R mouse model in NLRP3-deficient (NLRP3^-/-^) and wild-type (WT) mice. Following the targeted overexpression of USP25 in cardiomyocytes via administration of the AAV9-cTnT-Flag-USP25^oe^ vector in wild-type mice, alongside a control group treated with AAV9-cTnT-Empty Vector for a duration of 4 weeks, the mice were subjected to a 30-minute ischemic episode, followed by a reperfusion period lasting 4 hours. This experimental protocol was designed to induce I/R injury.

**
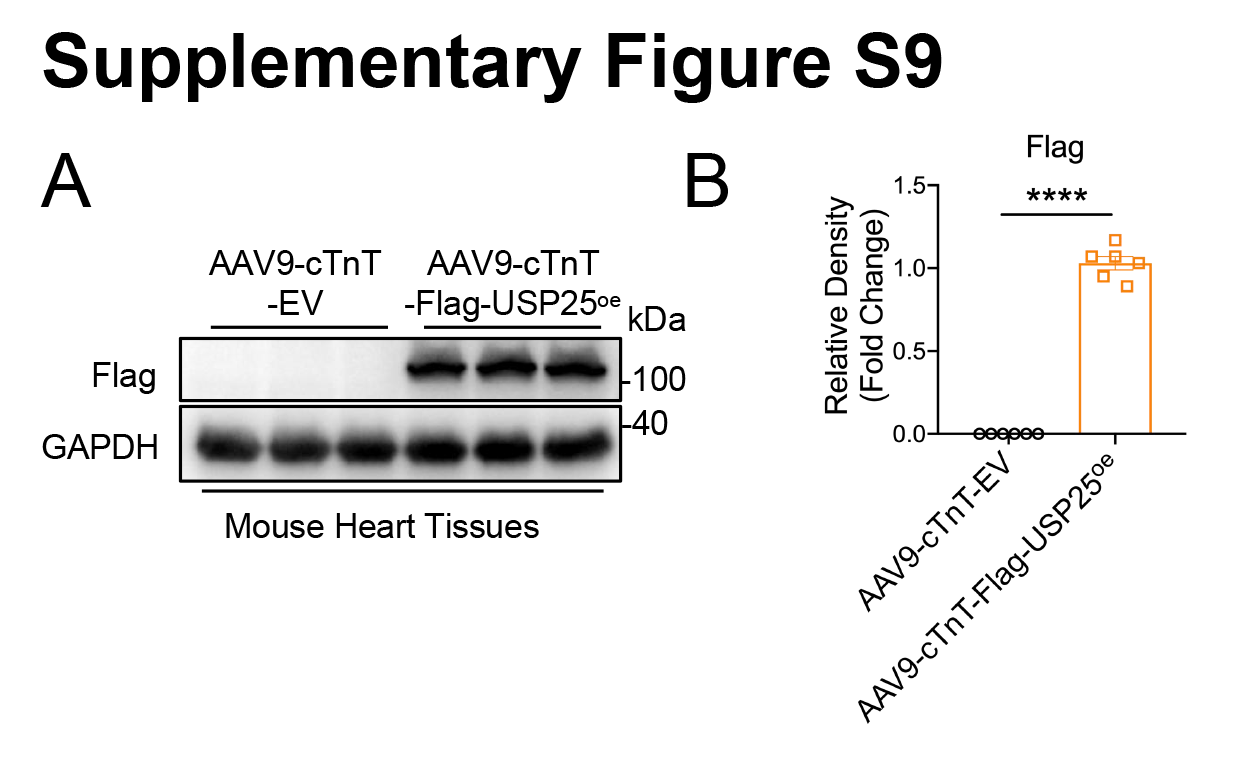
**

**Supplementary Figure S9:**

(**A-B**) Representative western blot results of Flag in mouse heart tissues infected with AAV9-cTnT-EC or AAV9-cTnT-USP25^oe^ (A) and quantitative data (B). P < 0.0001. n = 6.

**
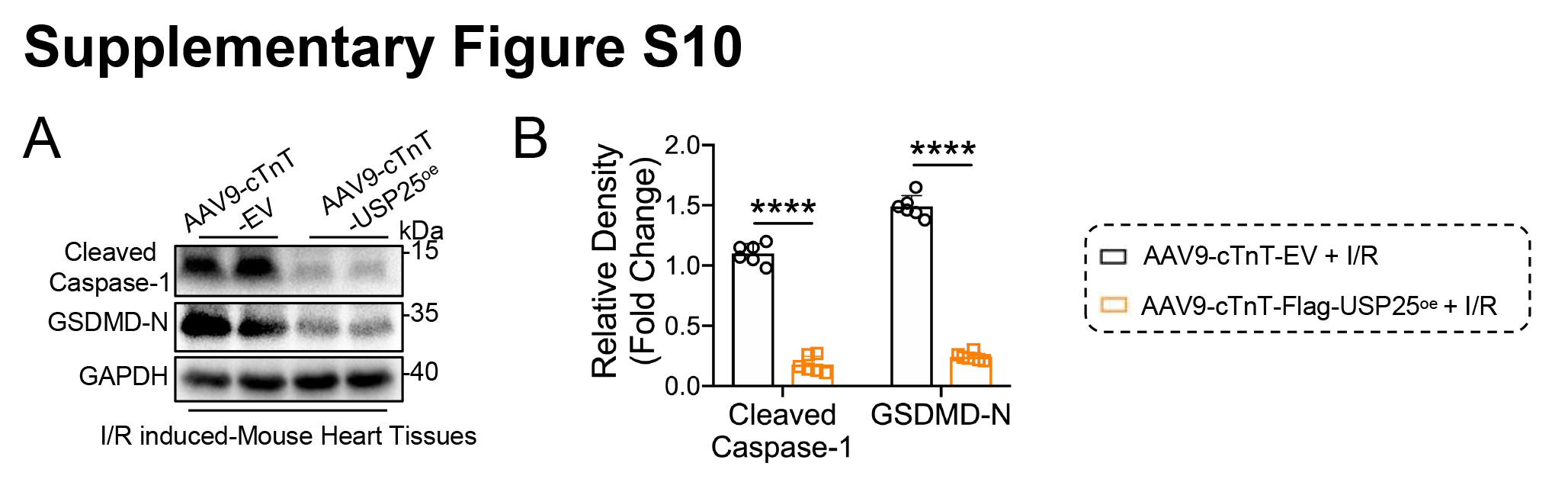
**

**Supplementary Figure S10:**

(**A-B**) Representative western blot results of Cleaved Caspase-1 and GSDMD-N in I/R-induced mouse heart tissues infected with AAV9-cTnT-EC or AAV9-cTnT-USP25^oe^ (A) and quantitative data (B). P < 0.0001. n = 6.

**
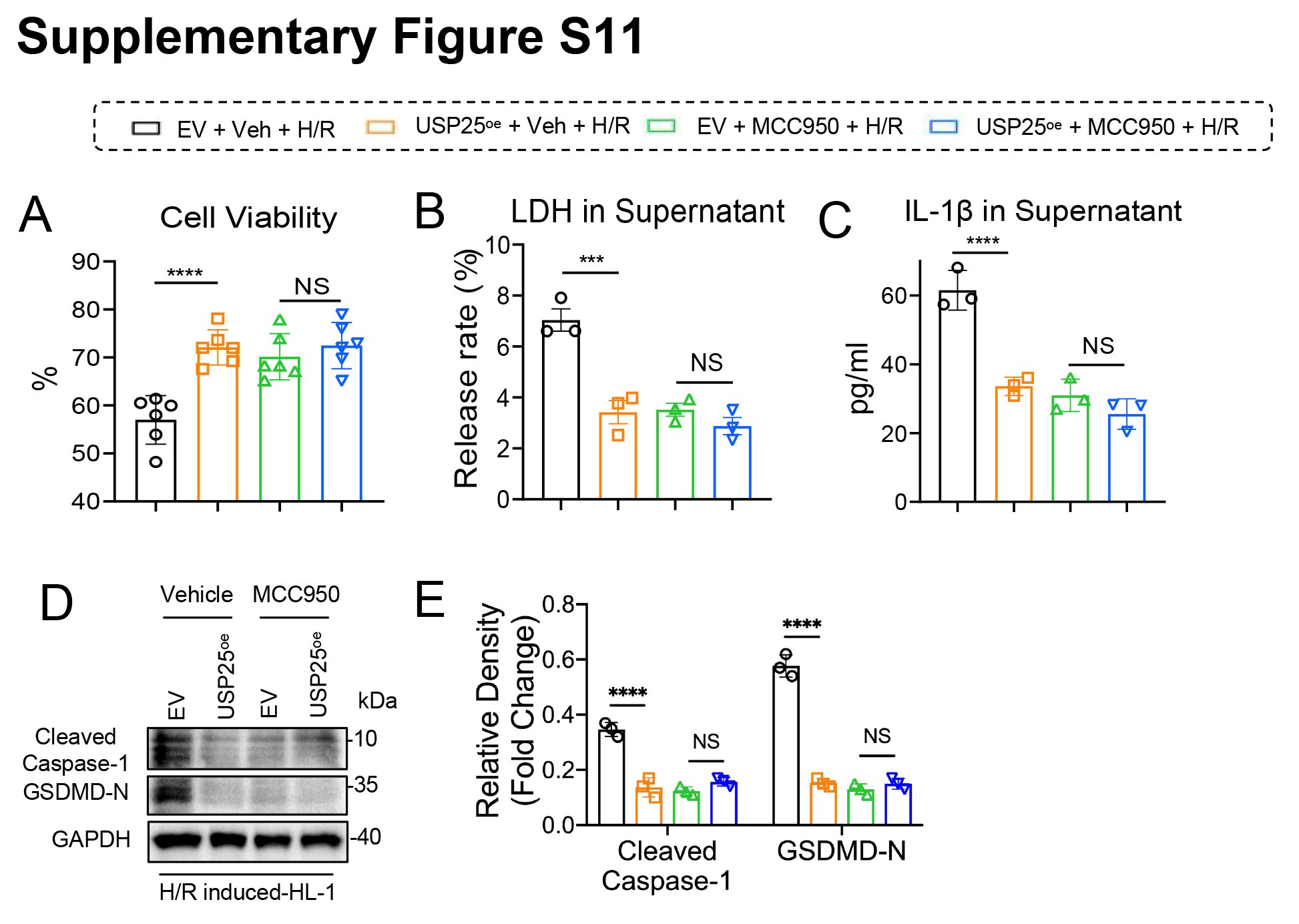
**

**Supplementary Figure S11:**

(**A**) Cell viability of HL-1. n = 6. **(B-C)** The release of LDH (B) and IL-1β (C) in HL-1. n = 3. (**E-F**) Representative western blot results of Cleaved Caspase-1 and GSDMD-N in I/R-induced HL-1 (E) and quantitative data (F). n = 3. NS, represents P > 0.05, ****, P < 0.0001.
